# Carbon-Heteroatom Bond Formation via Photoinduced Decarboxylative Radical Coupling Reactions

**DOI:** 10.1021/acsomega.5c08992

**Published:** 2025-11-17

**Authors:** Danilo F. C. de Benedicto, Giovanna S. Tâmega, Mateus O. Costa, Marco A. B. Ferreira, Ricardo S. Schwab

**Affiliations:** Laboratory of Synthesis, Catalysis, and Modeling (SintCatMol), Chemistry Department, 67828 Federal University of São Carlos (UFSCar), Rodovia Washington Luís, km 235 − SP − 310, São Carlos, São Paulo 13565-905, Brazil

## Abstract

Carboxylic acids are among the most widely used and versatile feedstock chemicals in organic synthesis. The generation of alkyl and aryl radicals through decarboxylation has emerged as a valuable strategy for the formation of both C–C and C–heteroatom bonds. Nevertheless, many reported protocols still rely on prior activation of carboxylic acids, which limits their cost-effectiveness and compromises environmental sustainability. This review aims to provide a comprehensive and critical overview of photoinduced decarboxylative coupling reactions employing free carboxylic acids as radical precursors for C–heteroatom bond formation. Particular emphasis is placed on the mechanistic insights underpinning these transformations, along with a discussion of their respective advantages, limitations, and potential for further development. By delineating the progress achieved thus far, we hope to stimulate continued innovation toward more sustainable, efficient, and broadly applicable decarboxylative coupling strategies.

## Graphical abstract

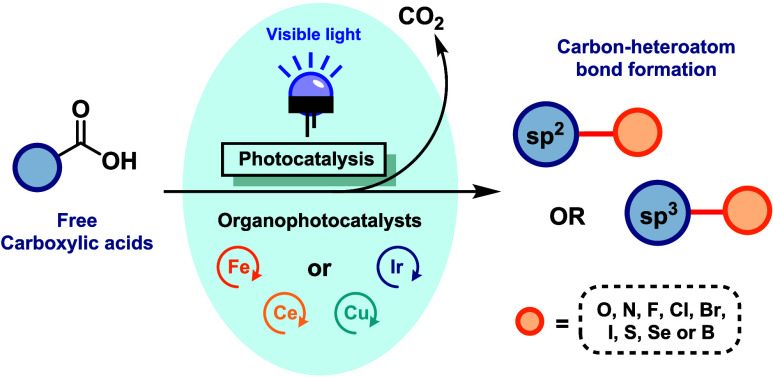

## Introduction

Over a century ago, Giacomo Ciamician, the first chemist to investigate light as an energy source to promote chemical transformations, predicted the rise of photochemistry as a valuable tool in organic synthesis.^1^ Indeed, in the past decade the field of photochemistry and photocatalysis applied to organic synthesis has emerged enormously, with a particular emphasis on visible-light-promoted reactions, due to the attractive properties of visible light such as low cost, selectivity, and safety.^2–6^ Furthermore, the cost-effectiveness of light-driven reactions also make them promise to scale-up processes for industrial application.^7^

Though photocatalytic pathways have been applied to a wide variety of reactions, substrates, and products, carboxylic acids remain as a key starting material for chemical transformations. Carboxylic acids bear a key position on chemical space due to its versatility, being one of the most structurally diverse substrates commercially available.^8^ Moreover, their stability makes them valuable coupling partners in several reactions to be applied in late-stage functionalization and medicinal chemistry.^9–11^ Carboxylic acids represent highly attractive starting materials in photochemical transformations, as their decarboxylation provides a straightforward and efficient entry point to alkyl and aryl radicals. Upon light absorption, carboxylic acids can perform single-electron-transfers (SETs), with formation of carboxyl radicals which spontaneously suffer decarboxylation to produce carbon-centered radicals.^9^

The generation of alkyl/aryl radicals through photoinduced decarboxylation usually requires a preactivation reaction that turns the free carboxylic acid into other more reactive compound (more commonly a phthalimide ester derivative or hypervalent iodine species, Scheme 1) that can absorb light and suffer further reactions.^12,13^ In this context, employing free (inactivated) carboxylic acids to perform decarboxylative alkyl/aryl radical generation presents the advantage of improving the atom economy and sustainability of the synthetic routes by diminishing the amount of reaction steps and, consequently, the amount of time and byproducts. Photoinduced decarboxylative reactions and other decarboxylative coupling methodologies have been covered in several recent review articles in a more general approach.^14–20^ In this review, we aimed to focus our attention on free carboxylic acids as alkyl/aryl radical sources in photoinduced radical coupling reactions to achieve carbon-heteroatom bond formation.

**Scheme 1:**
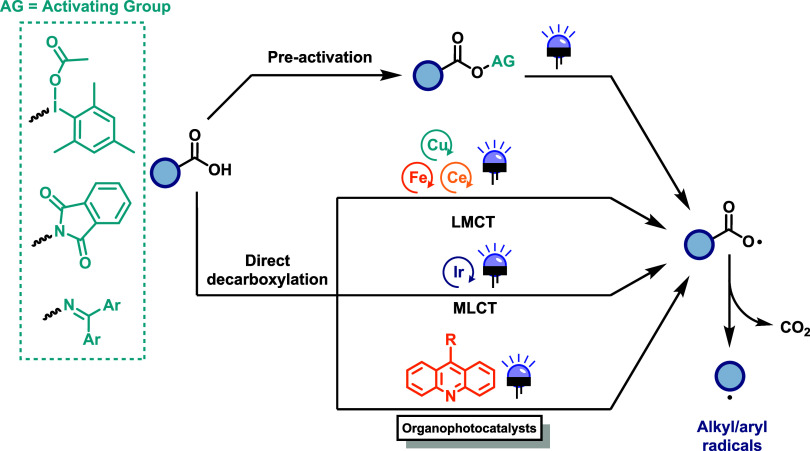
Photoinduced Decarboxylative Generation of Alkyl and Aryl Radicals

## Carbon-Oxygen Bonds

C–O bonds are found in fundamental organic functional groups such as ethers and alcohols, which represent indispensable motifs in synthetic chemistry. Accordingly, the development of methodologies for constructing carbon–oxygen (C–O) bonds remains a central focus for chemists. Conventional approaches to C–O bond formation, however, often rely on harsh conditions, stoichiometric reagents, or strong bases, thereby limiting their substrate scope and functional group tolerance. To overcome these challenges, alternative strategies have been developed, including decarboxylative methodologies that generate carbon-centered radicals capable of being intercepted by oxygen-based coupling partners.^21^ More recently, visible-light-driven decarboxylative approaches have attracted growing attention, opening new opportunities for C–O bond construction under milder and more sustainable conditions.

In the field of C–O bond formation via decarboxylative methods under visible light, several mechanistic pathways and reaction manifolds have been developed to access classes of substrates that are typically unreactive under polar or thermal conditions. Carboxylic acids were employed as radical precursors due to their commercial availability and synthetic utility. However, most existing methodologies utilizing this class of substrates are not selective for C­(sp^3^)–O bond formation and provide only a limited number of examples with narrow substrate scope. Moreover, oxidative decarboxylation strategies via photocatalysis typically rely on molecular oxygen as the oxygenation agent, leading predominantly to carbonylated products rather than C­(sp^3^)–O derivatives.^22,23^ In this context, Ritter and co-workers^22^ reported a radical decarboxylative hydroxylation of benzoic acids using copper-carboxylate LMCT (ligand-to-metal charge transfer) to access phenols (Scheme 2). This methodology circumvents the high activation barriers (∼30 kcal·mol^–1^) commonly associated with traditional transition-metal-mediated thermal decarboxylative carbometalation for C–C bond cleavage and C–O bond formation. This is possible because radical decarboxylation to form aryl radicals proceeds with significantly lower activation energies. To achieve this transformation, the authors employed photoinduced copper LMCT followed by carbometalation to generate arylcopper­(III) intermediates. The use of copper plays a critical role in suppressing undesired radical side reactions by rapidly capturing aryl radicals to form arylcopper­(III) species, which then undergo reductive elimination. A major challenge addressed in this study was the dual role of the substrate, which also functions as the oxygen-based nucleophile in the coupling step. Ideally, the nucleophilic carboxyl radical should decarboxylate at a slower rate than the substrate-derived carboxyl radical. To overcome this, the authors introduced an exogenous oxygen-based nucleophile capable of forming a carboxyl radical that undergoes faster back electron transfer (BET) or hydrogen atom transfer (HAT), thereby regenerating the corresponding carboxylic acid to serve as the nucleophile (Scheme 2). Thiophene-2-carboxylate (TC) was identified as the optimal coupling partner, as π-donation from the sulfur atom strengthens the C–COO^•^ bond, thereby retarding the rate of decarboxylation. The mechanism initiates with LMCT from the carboxylate ligand to Cu­(II) in the copper­(II) carboxylate complex upon visible light irradiation. This induces homolytic cleavage of the O–Cu­(II) bond, generating an aryloxyl radical that undergoes decarboxylation with CO_2_ extrusion to form an aryl radical which is rapidly trapped by copper. The aryl radical is intercepted either by the Cu­(II)–TC complex or the Cu­(I)–TC species and is then oxidized by Cu­(II) to form an arylcopper­(III)–TC intermediate. Reductive elimination affords the aryl–TC ester, which is hydrolyzed by LiOH to yield the final phenol product. The proposed mechanism is supported by UV–Vis spectroscopy and radical trapping experiment.

**Scheme 2:**
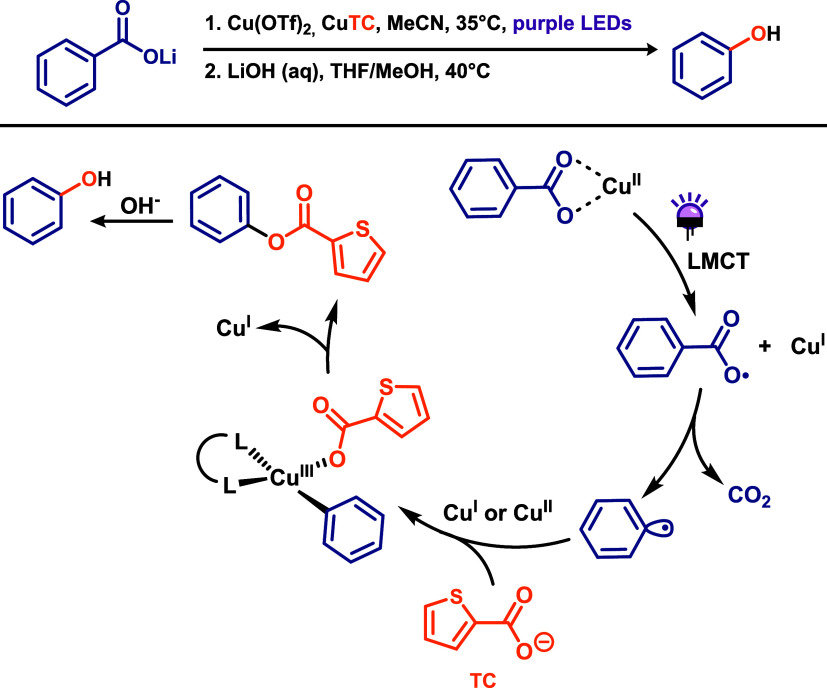
Decarboxylative Hydroxylation of Benzoic Acids

Still within the LMCT framework, but in a catalytic context, Denkler and co-workers^23^ reported an iron-catalyzed decarboxylative C­(sp^3^)–O bond formation under mild, base-free conditions with visible light irradiation (Scheme 3). The authors developed a direct iron-catalyzed decarboxylative cross-coupling of unactivated carboxylic acids using 2,2,6,6-tetramethylpiperidine-1-oxyl (TEMPO) as the oxygenation reagent, enabling an anaerobic decarboxylative oxygenation pathway. In addition to acting as an oxygen source, TEMPO plays a crucial dual role: as a masked base to deprotonate the carboxylic acid and (2) as an oxidant that regenerates Fe­(II) back to Fe­(III). Catalytic Fe­(OTf)_3_ complex was used as the iron source, and the ligand *N*,*N*-dimethyl-*N*,*N*-bis­(pyridin-2-ylmethyl)­ethane-1,2-diamine (L1) was found to be essential for catalytic activity. This methodology displayed broad functional group tolerance and accommodated a wide range of substrates, including those generating primary and secondary alkyl radicals, species typically prone to instability. Notably, structurally complex and bioactive molecules were successfully functionalized, underscoring the potential for late-stage modification. The synthetic versatility of the TEMPO-functionalized products was further demonstrated through various postfunctionalization transformations. To understand the mechanism, a series of experiments were conducted. Kinetic studies identified the rate-determining step (RDS) as the photoinduced decarboxylative generation of the alkyl radical from the Fe­(III) dicarboxylate complex. A central mechanistic question concerned the reoxidation of Fe­(II) to Fe­(III) by TEMPO. Cyclic voltammetry experiments indicated that direct oxidation of [(L1)­Fe^II^(OCOR)_2_] by neutral TEMPO is thermodynamically unfavorable, suggesting that TEMPO^+^ (the oxoammonium cation) is likely responsible for the reoxidation process. DFT calculations supported this conclusion, identifying the most exergonic pathway as a single-electron transfer (SET) from [(L1)­Fe^II^(OCOMe)_2_] to TEMPO^+^. Additional experiments including chronoamperometry, EPR spectroscopy, and resting-state analysis confirmed that TEMPO plays three essential roles acting as (1) radical trap, (2) oxidant, and (3) internal base for carboxylic acid deprotonation. The proposed mechanism (Scheme 3) begins with complex (A), [(L1)­Fe^III^(OCOR)­(MeCN)]^2+^, undergoing ligand substitution with an additional equivalent of carboxylate to form complex (B), [(L1)­Fe^III^(OCOR)_2_]^+^. Upon photoexcitation, LMCT from the carboxylate to Fe­(III) triggers cleavage of the Fe–OCOR bond and extrusion of CO_2_. This step, identified as the RDS, generates the alkyl radical and complex (C), [(L1)­Fe^II^(OCOR)­(MeCN)]^+^. Subsequently, SET with TEMPO^+^ oxidizes complex (D), closing the catalytic cycle.

**Scheme 3:**
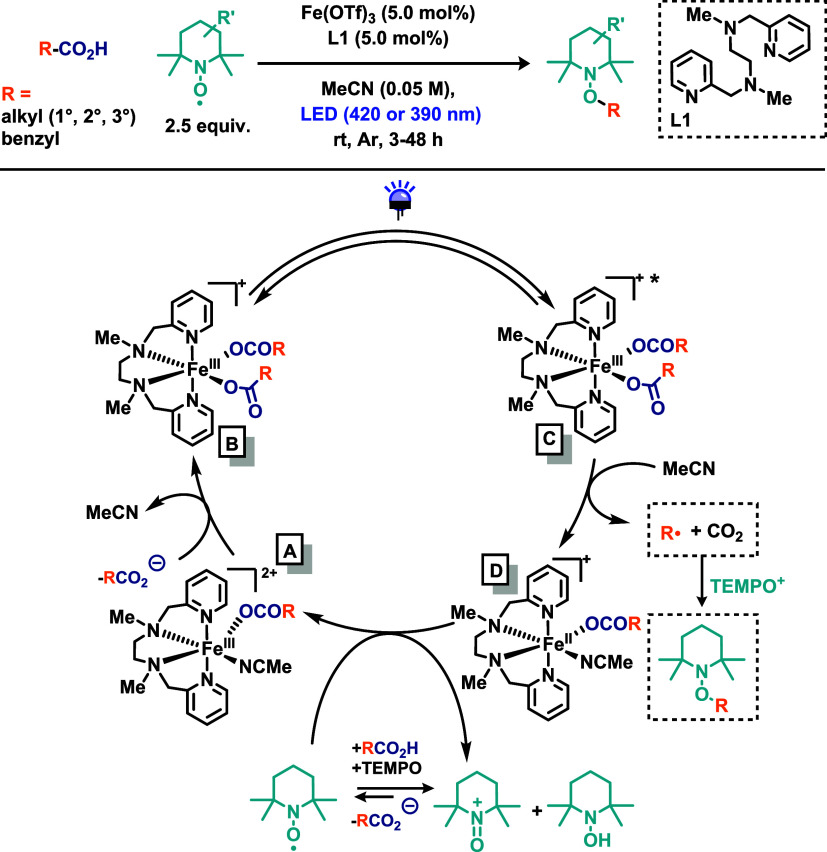
Iron-Catalyzed Decarboxylative C­(sp^3^)–O Bond Formation

Using a similar approach, Innocent and co-workers^24^ reported an example a C–O bond-forming via iron-mediated LMCT, notably carried out in the absence of ligands (Scheme 4). The reaction conditions closely resembled those described in the previous example, except for the use of FeBr_2_ as the iron source in a ligand free strategy. The methodology demonstrated a broad substrate scope, encompassing primary, secondary, and tertiary alkyl groups, as well as aryl substrates. The proposed mechanism relies on a similar concept previously explored, with the distinction that TEMPO^–^ is suggested to be generated, thereby enabling the regeneration of the Fe­(III) catalyst. Consequently, TEMPO is expected to act both as an oxidant and as a base, facilitating the deprotonation of the carboxylic acid. Additionally, the robustness of the method was demonstrated by increasing the reaction scale from 0.3 to 5 mmol of carboxylic acid, achieving similar yields after 48 h. The synthetic utility of the resulting alkoxyamine products was further explored. Reduction of these intermediates afforded the corresponding alcohols, whereas oxidation reactions provided access to aldehydes and ketones. Additional derivatizations were also demonstrated, including deuteration, fluorination, and C–C bond formation using iridium catalysts, affording the desired products in moderate yields.

**Scheme 4:**
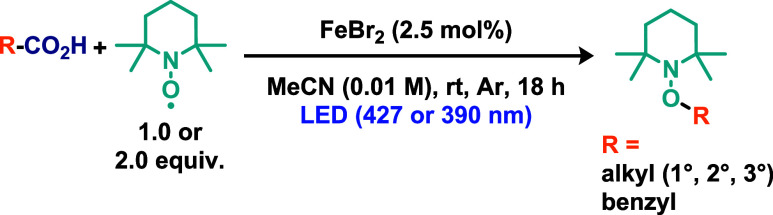
C–O Bond Formation via Iron-Mediated LMCT in the Absence of Ligands

In another study Innocent and co-workers^25^ applied the iron-catalyzed, photoinduced decarboxylative strategy to the synthesis of carbonyl derivatives targeting aldehydes and ketones derived from primary and secondary carboxylic acids (Scheme 5). Among the tested conditions, the best performance was achieved using catalytic amounts of Fe­(NH_4_)_2_(SO_4_)_2_·6H_2_O. Control experiments confirmed that both the iron catalyst and light irradiation were essential for the transformation. With respect to the substrate scope, the methodology exhibited high functional group tolerance and proved especially effective for the late-stage derivatization of active pharmaceutical ingredients (APIs). In general, according to the authors, aldehydes were obtained in lower yields than ketones likely due to the volatility of some benzaldehyde products and their propensity to oxidize into benzoic acids under aerobic conditions. In this system, molecular oxygen acts as the oxidant, converting the Fe­(II) precatalyst into a carboxylate Fe­(III) complex. Additionally, the method enabled the formation of amides using *N*-protected amino acids as precursors, showcasing its potential for peptide modification through selective C-terminal functionalization. Mechanistic investigations identified iron carboxylate complexes by IR spectroscopy, and UV–Vis spectroscopy further suggested that the presence of a ligand may not be essential for the visible-light-induced homolytic cleavage of the Fe–O bond, although the ligand may act as a base by facilitating carboxylic acid deprotonation. Based on previous reports indicating that alkyl hydroperoxides can serve as intermediates in decarboxylative oxidations, the authors tested these compounds directly as substrates. Interestingly, under these conditions, neither an O_2_ atmosphere nor light irradiation was required to generate the ketone product, suggesting the involvement of hydroperoxides as transient intermediates. Furthermore, light on/off experiments demonstrated that the reaction does not proceed via a radical chain mechanism. The proposed mechanism (Scheme 5) begins with the *in situ* formation of a carboxylate–Fe­(III) complex (A), mediated by molecular oxygen. Upon photoexcitation, this complex undergoes ligand-to-metal charge transfer (LMCT), resulting in homolytic cleavage of the Fe–O bond and formation of a Fe­(II) complex (D) and a carboxyl radical (B). This radical then undergoes decarboxylation to yield an alkyl or benzyl radical, which can be trapped by O_2_ to form a peroxyl radical intermediate (C), which may follow three pathways to afford the product. The first possibility is the recombination of radical (C) with the Fe­(II) carboxylate (D) to deliver intermediate (E). A subsequent ligand exchange with the carboxylic acid regenerates the photoactive Fe­(III) complex (A) and gives an alkyl hydroperoxide (F), which furnishes the final product after dehydration. Alternatively, intermediate (C) can evolve into the corresponding α-hydroperoxyl radical (G). This radical can eliminate an OH^•^ radical, affording the final carbonyl product. Moreover, Intermediate (C) can also abstract a hydrogen atom from an external H donor to form the intermediate (F), which generates the product by dehydration. Finally, it was shown that ligand L1 does not play a crucial role in the photoinduced process, but instead acts as a base, assisting in the deprotonation of the carboxylic acid.

**Scheme 5:**
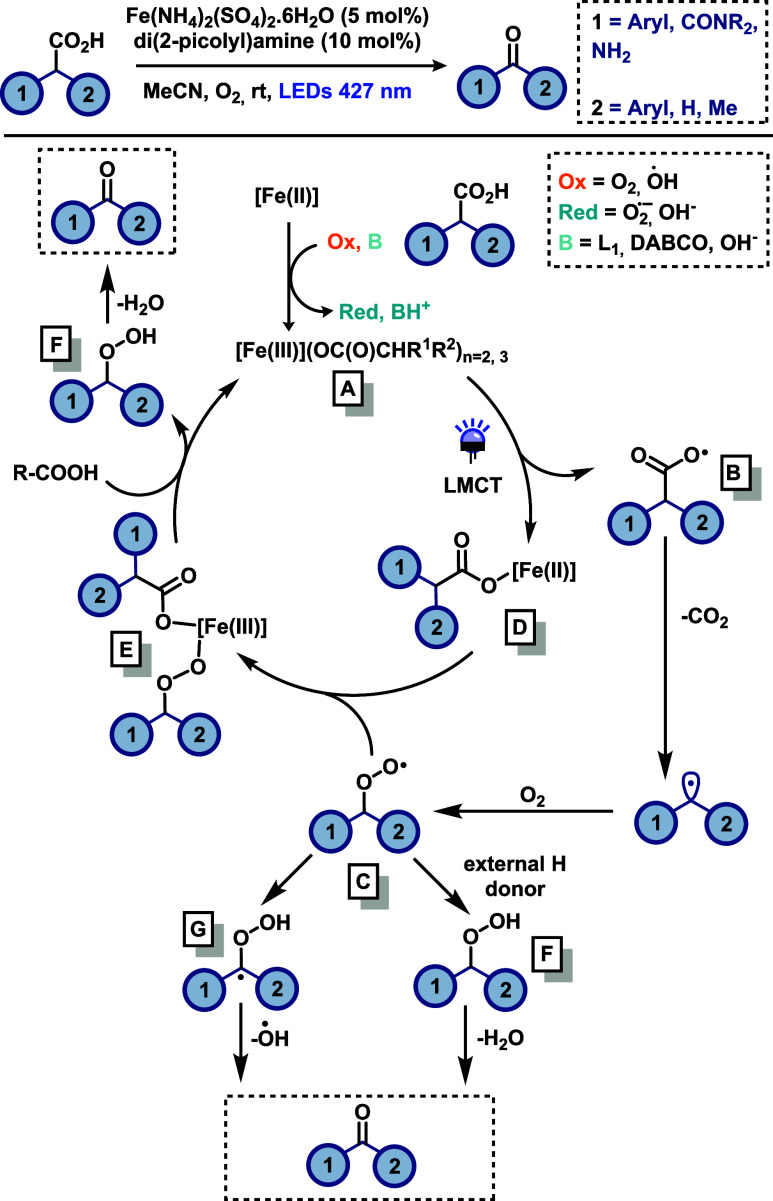
Iron-Photocatalyzed Decarboxylative Synthesis of Carbonyl Derivatives

Another example with Fe­(III)-mediated LMCT reactivity involving unactivated carboxylic acids was reported by Lutovsky and co-workers,^26^ who employed a stoichiometric and more general approach enabling both C–C and C–heteroatom coupling, including multiple cases of C–O bond formation. Using 4-dimethylphenylacetic as the model substrate and alcohols as coupling partners (Scheme 6) the authors demonstrated a broad scope of etherification reactions. Following a similar LMCT-based mechanistic pathway, a variety of carboxylic acids and alcohols were successfully coupled to afford diverse ethers, including the functionalization of active pharmaceutical ingredients (APIs). Notably, the protocol was also directly applicable to thioetherification reactions, enabling the formation of C–S bonds under analogous conditions.

**Scheme 6:**
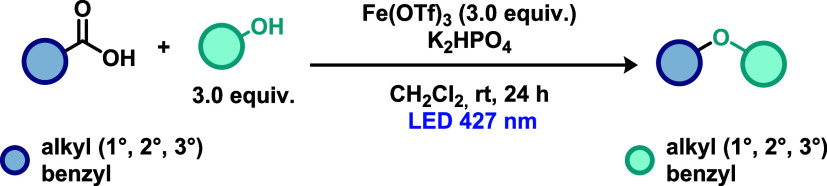
Iron-Catalyzed Decarboxylative Synthesis of Ethers

Khan and co-workers^27^ described a C–O bond formation strategy involving the decarboxylative hydroxylation of carboxylic acids using an iridium-based photocatalyst (Scheme 7). This type of catalyst is well-known for its ability to promote electron transfer via a metal-to-ligand charge transfer (MLCT) mechanism, in contrast to the ligand-to-metal charge transfer (LMCT) pathways described in the examples above. The protocol enabled the efficient synthesis of alcohols derived from primary, secondary, and tertiary phenylacetic acids, as well as from aliphatic carboxylic acids, affording the corresponding products in excellent yields. The proposed mechanism initiates with a photocatalytic decarboxylation, in which visible-light irradiation promotes excitation of the Ir­(III) complex via an MLCT pathway. The excited complex then engages in a single electron transfer (SET) with the deprotonated carboxylic acid, generating an alkyl or aryl radical species through decarboxylation. This radical subsequently reacts with molecular oxygen to form a peroxyl radical, which is rapidly reduced by NaBH_4_ to furnish the corresponding alcohol product. Moreover, the authors demonstrated the scalability of the methodology by performing the reaction on a gram scale.

**Scheme 7:**
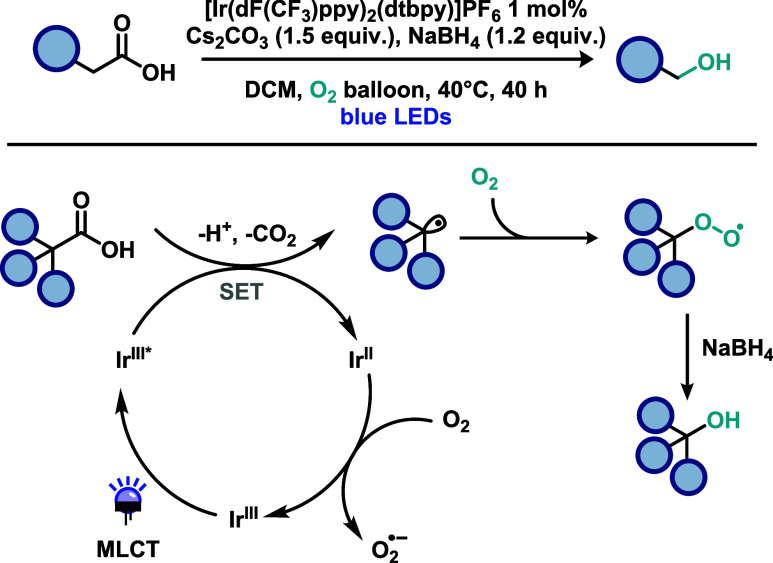
Ir-Catalyzed Decarboxylative Hydroxylation of Carboxylic Acids

Beyond metal-based strategies, C–O bond formation from carboxylic acids has also been investigated using metal-free approaches, particularly those involving organic photocatalysts. In this context, Song and co-workers^28^ reported the first example of a photocatalytic, direct decarboxylative hydroxylation of carboxylic acids (Scheme 8). This method employed molecular oxygen (O_2_) as a greener oxidant, in contrast to previous strategies that rely on high-valent metal oxidants. The underlying hypothesis was that O_2_ could act as a radical trap, capturing the intermediate generated through decarboxylation of the carboxylic acid. Using an acridinium-based organic photocatalyst the authors efficiently synthesized various alcohols from a broad range of substrates, including drug-like molecules derived from nonsteroidal anti-inflammatory drugs (NSAIDs). To probe the reaction mechanism, the authors investigated whether O_2_ was indeed responsible for trapping the carboxylic acid-derived radical to furnish the alcohol product. To identify the oxygen source in the product, ^18^O-labeling experiments were performed. When ^18^O-labeled O_2_ was used, the corresponding ^18^O-labeled alcohol was obtained. In contrast, when ^18^O-labeled H_2_O was employed under identical conditions, no isotope incorporation was observed. The involvement of singlet oxygen was further examined through the addition of DABCO, a well-established quencher of this species. Since the reaction proceeded unaffected, this hypothesis was ruled out. The methodology was also successfully scaled up, and natural sunlight was evaluated as the light source, affording the alcohol product in excellent yield. Mechanistically, the process closely parallels the iridium-based system described earlier: following photoexcitation of the acridinium photocatalyst, a SET step oxidizes the carboxylate anion, generating the key radical intermediate via decarboxylation. This radical is subsequently trapped by molecular oxygen to form a peroxyl radical, which is then reduced (e.g., by NaBH_4_) to furnish the corresponding alcohol. Notably, oxygen plays a dual role in this transformation, acting both as the radical trap and as the oxidant that regenerates the photocatalyst.

**Scheme 8:**
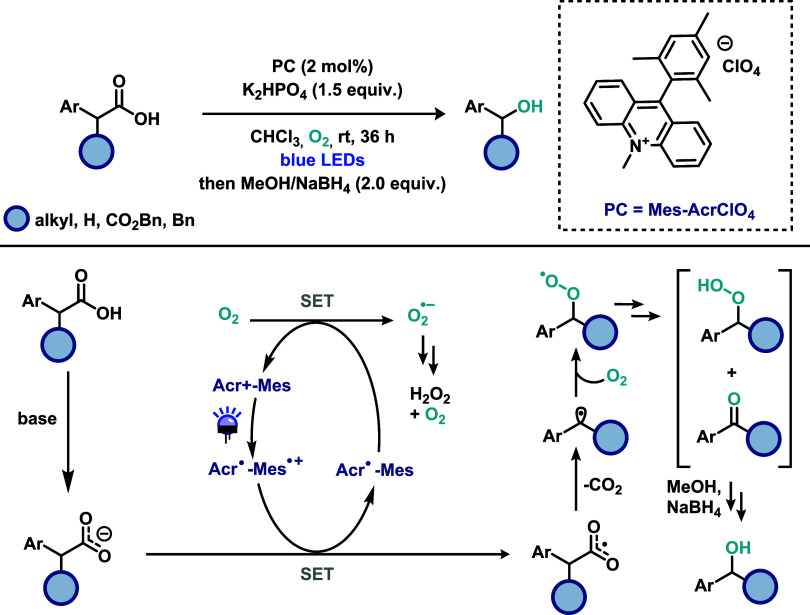
Metal-Free Direct Decarboxylative Hydroxylation of Carboxylic Acids

Following Song’s seminal work, other organophotocatalysts have been explored for decarboxylative transformations. He and co-workers^29^ reported the decarboxylation of arylacetic acids to access aldehydes and ketones using 4-CzIPN as a metal-free photocatalyst, notably under ambient conditions with molecular oxygen as the terminal oxidant (Scheme 9). The photocatalyst was employed in low loading (1 mol %), and 1,1,3,3-tetramethylguanidine (TMG) was identified as the optimal base for the transformation. A diverse range of carboxylic acids was investigated, including heteroaromatic derivatives. However, meta-substituted phenylacetic acids afforded poor yields. On the other hand, α-substituted aromatic acetic acids were efficiently converted into the corresponding ketones. In contrast, aliphatic carboxylic acids were found to be unsuitable under the reaction conditions, highlighting the limitations in substrate scope. Mechanistic investigations supported a radical pathway. Photoluminescence quenching experiments demonstrated that molecular oxygen effectively quenches the excited state of 4-CzIPN. Additionally, the addition of *p*-benzoquinone, a known scavenger of superoxide (O_2_^•^) radicals, significantly diminished product formation, suggesting the critical involvement of this reactive oxygen species. These observations were further corroborated by electron paramagnetic resonance (EPR) experiments. Based on these results, the authors proposed the following mechanism: upon visible-light irradiation, 4-CzIPN is photoexcited, and the resulting excited state undergoes single-electron transfer (SET) with molecular oxygen generating the superoxide radical (O_2_^•^) and the oxidized form of photocatalyst. This oxidized 4-CzIPN species then oxidizes the carboxylate anion, promoting decarboxylation and formation of a benzylic radical. The resulting radical recombines with superoxide radical to form an anionic peroxide intermediate, which is subsequently protonated and undergoes dehydration to furnish the desired carbonyl product.

**Scheme 9:**
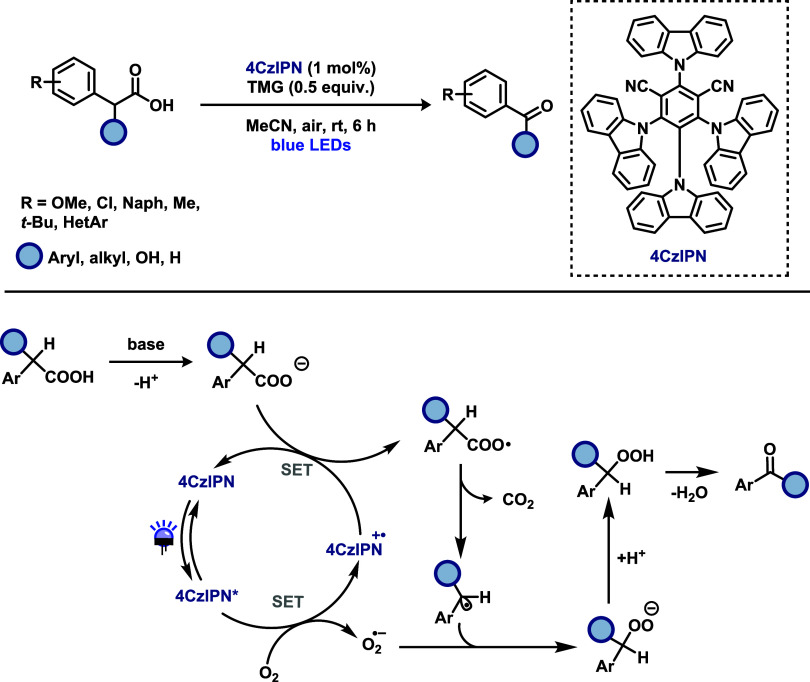
Metal-Free Decarboxylation of Arylacetic Acids to Access Aldehydes and Ketones

More recently, Lou and co-workers^30^ addressed the challenge of synthesizing organic peroxides through a decarboxylative peroxidation strategy using *tert*-butyl hydroperoxide (TBHP), under photocatalysis conditions with an acridinium-based organophotocatalyst (Scheme 10). A wide range of arylacetic acids, including drug-like molecules, was successfully employed in this transformation. Moreover, the reaction was scaled up efficiently, and the resulting organic peroxide was subjected to further derivatization, highlighting the synthetic utility of the method. The proposed mechanism closely resembles those described in the previous examples, with the key difference that TBHP serves a dual role: oxidant responsible for regenerating the photocatalyst and as the radical coupling partner. Upon photoexcitation of the acridinium catalyst, the carboxylate anion undergoes single-electron oxidation, leading to decarboxylation and formation of a benzyl radical. The reduced form of the photocatalyst then reacts with TBHP, regenerating its ground-state and producing the tert-butoxy radical. This tert-butoxy radical abstracts a hydrogen atom from another molecule of TBHP, generating a peroxyl radical, which subsequently couples with the benzyl radical to afford the desired organic peroxide product.

**Scheme 10:**
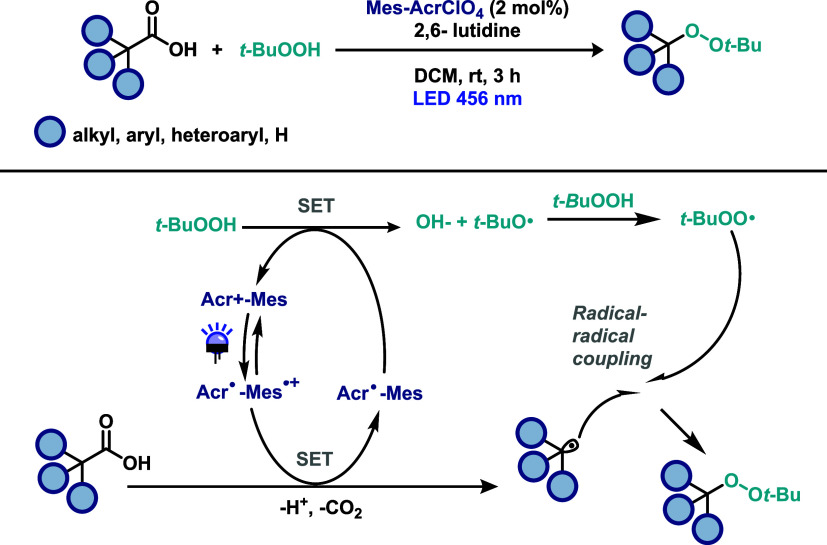
Decarboxylative Peroxidation of Carboxylic Acids

Extending the scope of metal-free methodologies, another emerging photocatalytic strategy involves the use of photoactive materials as heterogeneous catalysts. Murugesan and co-workers^31^ demonstrated the synthesis of carbonyl compounds using mesoporous graphitic carbon nitride (mpg-CN) as a heterogeneous photocatalyst (Scheme 11). This heterogeneous photocatalytic system exploits the favorable properties of mpg-CN, including high stability, large surface area, recyclability, and a suitable band gap, which exhibits properties comparable to many homogeneous photocatalysts. This study represents the first example of mpg-CN applied to this class of transformation and introduces a general strategy for converting structurally diverse substrates into carbonyl compounds. The scope encompassed primary and secondary benzylic acids, as well as aliphatic acids. Importantly, the method displayed good tolerance, including compatibility with heterocyclic acids and acid-containing pharmaceutical compounds. Moreover, the strategy enabled the synthesis of amides from amino acids in good yields, further demonstrating its versatility. Regarding catalyst recyclability, mpg-CN could be reused up to five times with minimal loss of photocatalytic activity. To evaluate its performance and stability, a series of tests were conducted, including mechanistic studies. Oxygen suppression experiments confirmed the essential role of molecular oxygen in product formation, and the use of TEMPO as a radical scavenger supported a radical-based reaction pathway. The proposed mechanism begins with the excitation of mpg-CN under visible-light irradiation, generating an oxidative site (hole, h^+^) in the valence band (VB) and a reductive site (electron) in the conduction band (CB). Molecular oxygen is reduced by electrons in the CB to form a superoxide radical anion, while the substrate is oxidized by VB holes to generate an alkyl or aryl radical. This radical then reacts with O_2_ to form a peroxide intermediate, which undergoes a hydrogen atom transfer (HAT) to produce a peroxyl radical. Finally, the carbonyl product is formed via elimination of a hydroxyl radical.

**Scheme 11:**
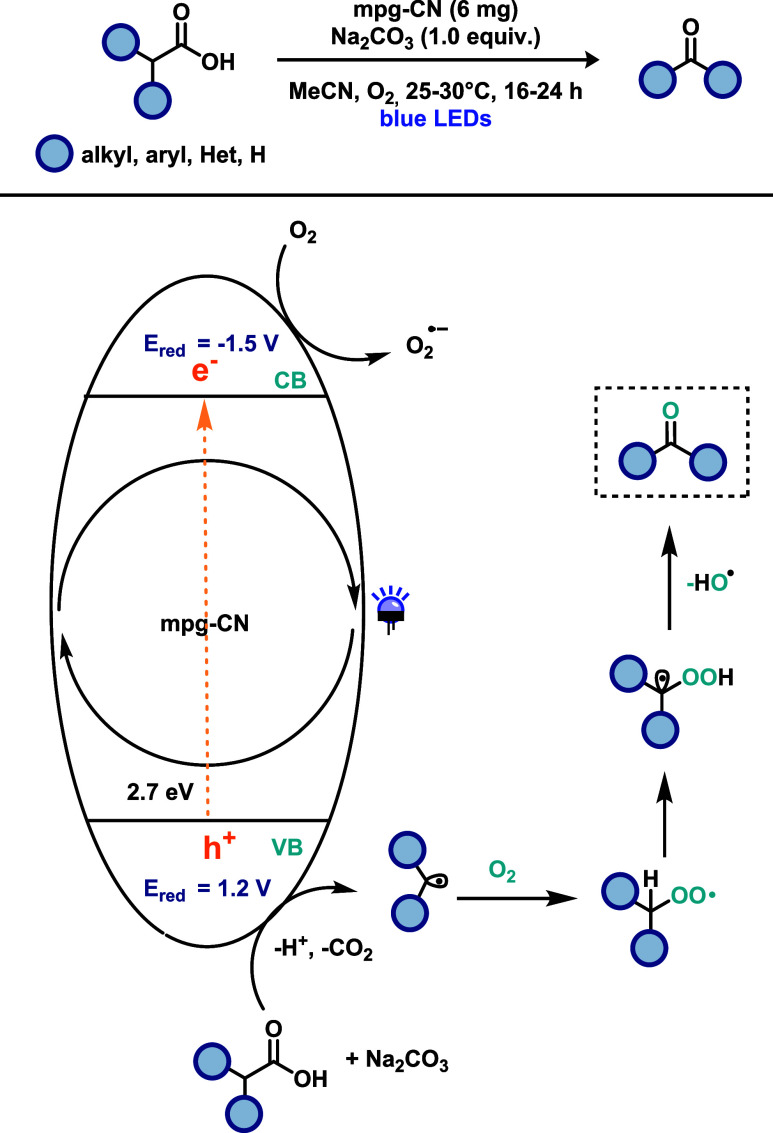
Carbon Nitride (mpg-CN)-Catalyzed Synthesis of Carbonyl Compounds

Using a similar approach, Zhu and co-workers^32^ reported a metal-free decarboxylative oxygenation of α-amino acids to form amides via heterogeneous photocatalysis (Scheme 12). In a detailed catalyst optimization, the authors aimed to develop a methodology for amide formation that operates under base-free and metal-free conditions. Specifically, by evaluating a variety of carbon nitride photocatalysts, they identified CN-600 as the optimal material for this transformation. Under visible-light irradiation and using molecular oxygen as the sole oxidant, the method enabled the conversion of eight different α-amino acids into their corresponding amides under mild and environmentally benign conditions. The C=O bond formation follows a mechanism analogous of that shown in Scheme 11. This work highlights the potential of heterogeneous carbon nitride photocatalysts to promote selective oxidative transformations without reliance on transition metals, offering a sustainable alternative for amide bond synthesis.

**Scheme 12:**
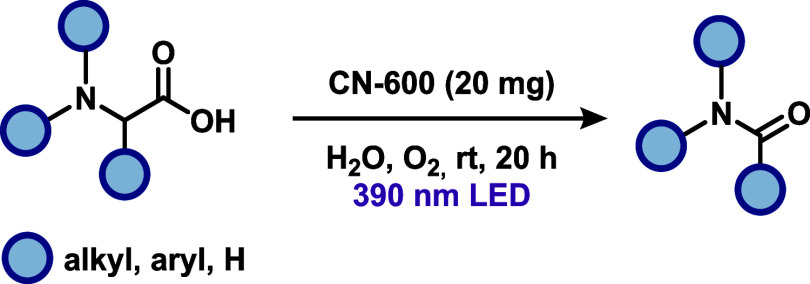
Carbon Nitride-Catalyzed Synthesis of Amides

## Carbon-Nitrogen Bonds

Many naturally occurring organic compounds contain at least one nitrogen atom, and nitrogen-containing molecules have wide applications in the synthesis of pharmaceuticals, functional materials, and agrochemicals. Because of the central role of nitrogen in determining the chemical properties and reactivity of these compounds, the development of efficient and innovative C–N bond-forming protocols has become a major area of research.^33^ Although numerous synthetic methods for C–N bond formation have been reported over the past decades, decarboxylative and, more recently, radical decarboxylative approaches have emerged as particularly powerful strategies.^19,33^

Li and Zeng reported in 2023 the first example of a dual catalytic system involving both iron and copper for C–N bond formation.^34^ In this strategy, Fe­(III) generates alkyl radicals from carboxylic acids via (LMCT) mechanism, under 390 nm LED irradiation (Scheme 13). Concurrently, Cu­(II) operates through a two-electron redox pathway: the amine, upon deprotonation in the presence of DBU, coordinates to Cu­(II), forming intermediate (A). This species captures the LMCT-generated alkyl radical to form an alkyl–Cu­(III) intermediate (B). Reductive elimination from (B) then furnishes the C–N bond, yielding the final product. The catalytic cycle is closed by reoxidation of Cu­(I) to Cu­(II) using di*tert*-butyl peroxide (DTBP) as the terminal oxidant. This cross-coupling strategy demonstrated broad substrate scope, affording excellent yields with primary, secondary, and benzylic carboxylic acids in combination with aromatic amines. However, the methodology displayed limited efficiency with tertiary carboxylic acids (with the exception of the adamantyl derivative), as well as with aliphatic amines, amides, and other nitrogen-containing substrates. Notably, this dual catalytic system also enabled the conversion of amino acids into enamines via β-hydride elimination. In this transformation, the nitrogen atom adjacent to the radical center stabilizes the alkyl-Cu intermediate (C) formed after decarboxylation, suppressing undesired amine coordination. The decarboxyolefination was successfully achieved with *N*-protected, *N*-amidated, and *N*-aryl amino acid derivatives, highlighting the method’s versatility in enamine synthesis.

**Scheme 13:**
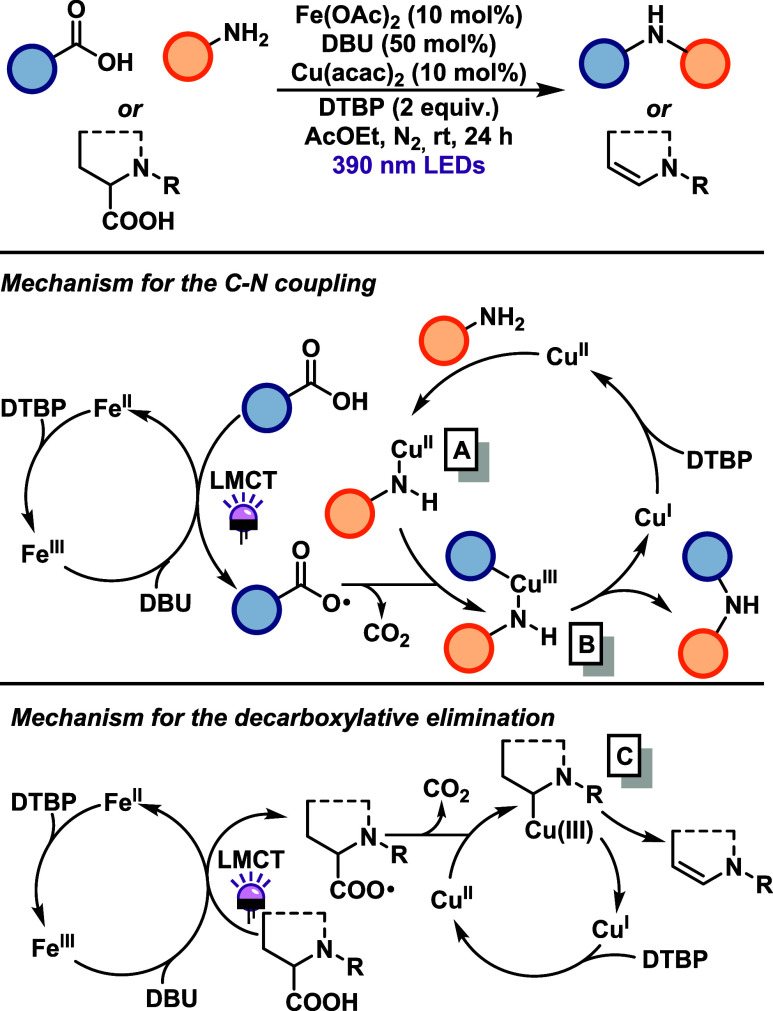
Iron–Copper-Dual-Catalytic System for C–N Bond Formation

In 2019, Larionov and co-workers^35^ reported a photoinduced proton-coupled electron transfer (PCET) mechanism enabling the direct decarboxylative alkylation (DDA) reaction, in which a Cu­(II) catalyst provided a C–N coupling through aromatic and heteroaromatic amines and alkyl precursors derived from carboxylic acids, even under aerobic conditions (Scheme 14). The methodology exhibits a broad substrate scope, including primary, secondary and adamantyl carboxylic acids, as well as alkylation of diarylamines, dual alkylation of anilines and heteroaromatic amines, although it shows limited tolerance to diverse functional groups. Additionally, the authors demonstrated a strategy for *N*-trideuteromethylation using AcOH-*d*_4_ as the deuterium source and achieved one-step synthesis of cyclic anilines employing 5-chlorovaleric acid as the alkylating agent. A detailed mechanistic proposal was developed based on DFT calculations and experimental studies. The proposed mechanism begins with coordination of the amine to Cu­(hfac)_2_, forming intermediate (A). Simultaneously, an acridine-based photocatalyst, upon 400 nm irradiation, mediates a PCET process that generates an alkyl radical via decarboxylation. This radical is trapped by (A) to form a Cu­(III) intermediate (B). Reductive elimination from (B) furnishes an anilinium intermediate, which undergoes a second PCET event and a hydrogen atom transfer (HAT) step, assisted by di*tert*-butyl peroxide (DTBP), to yield the final alkylated amine. During this step, Cu­(I) is reoxidized to Cu­(II), completing the catalytic cycle. Additionally, an alkoxide radical generated in this process is proposed to be responsible for regenerating the acridine photocatalyst.

**Scheme 14:**
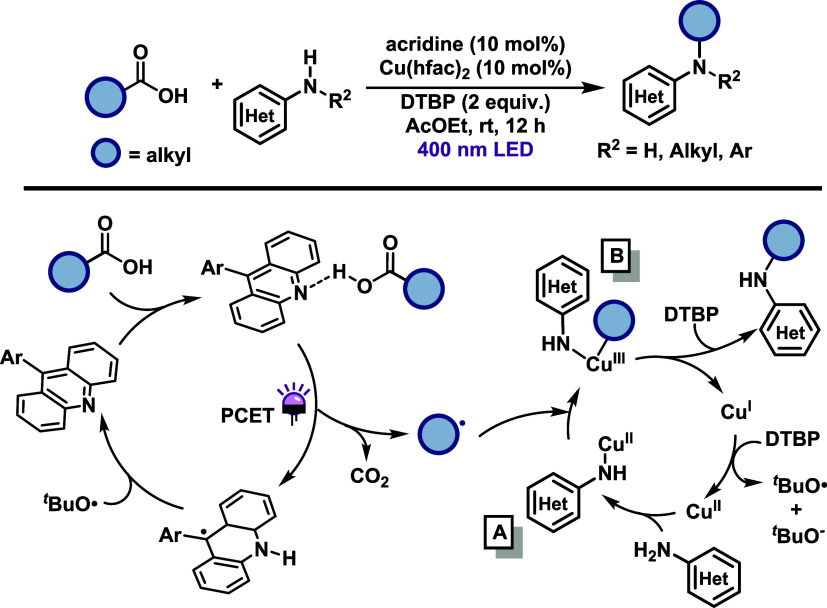
Direct Decarboxylative Alkylation of Amines

Yoon and co-workers developed an iron-mediated cross-nucleophile coupling that enables C–C, C–O, C–N and C–S bond formation via a radical-polar crossover pathway^26^ (Scheme 15). The method exhibits broad nucleophile scope, including electron-rich and some electron-poor arenes, alcohols, and sulfonamides, and tolerates a variety of functional groups such as halides, esters, ketones, and heterocycles. However, the reaction shows poor efficiency with basic aliphatic amines, likely due to their strong coordination to Fe­(III), which inhibits catalyst turnover. To address this limitation, the authors proposed a modified protocol in which the nucleophile is added after irradiation of the carboxylic acid in the presence of Fe­(III), allowing the cross-coupled product to be formed. Despite its versatility, the protocol requires stoichiometric amounts of FeCl_3_ and high nucleophile loadings (up to 50 equiv), which may limit its scalability and practical application. The proposed mechanism involves initial Fe­(III) mediated photodecarboxylation via LMCT process, generating an alkyl radical from the carboxylate precursor. This radical undergoes chlorine atom transfer from an Fe­(III)-chloride complex to form an alkyl chloride intermediate, which is then activated by Fe­(III) as a Lewis acid to undergo nucleophilic substitution, affording the desired product.

**Scheme 15:**
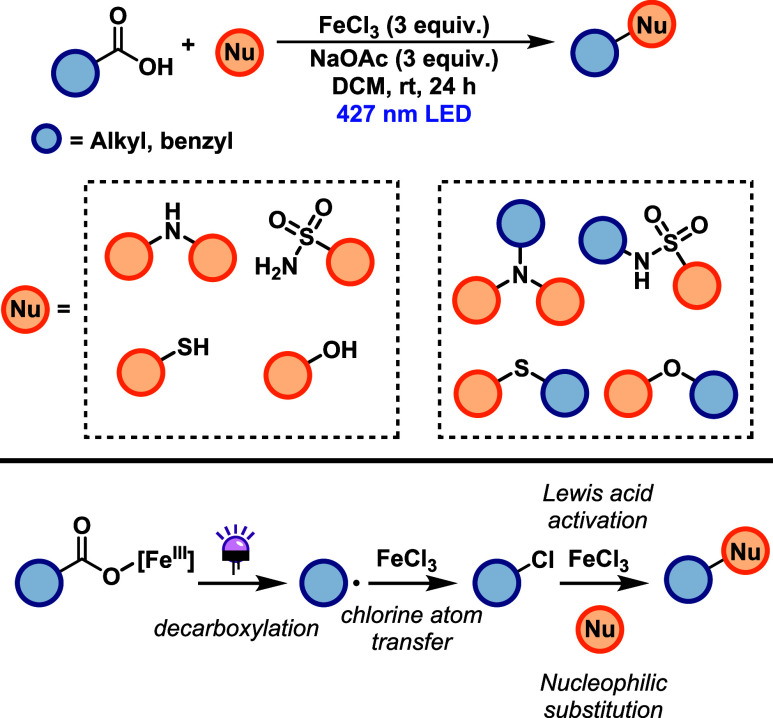
Iron-Mediated Cross-Nucleophile Coupling for C–O, C–N and C–S Bond Formation

West and co-workers^36^ reported an iron-catalyzed azidation of carboxylic acids that integrates ligand-to-metal charge transfer (LMCT) with radical ligand transfer (RLT) catalysis (Scheme 16). This protocol, based on simple iron nitrate salts and azidotrimethylsilane, allows direct azide incorporation into both activated and unactivated carboxylic acids without an external oxidant, with the nitrate counterion acting as the intrinsic oxidizing species. The protocol demonstrates broad functional group tolerance across various arylacetic acids, including electron-rich, electron-poor, and sterically hindered substrates. Notably, the reaction also accommodates unactivated primary and secondary aliphatic acids, substrates previously inaccessible to copper-based LMCT/RPC systems, albeit with generally lower efficiency. Mechanistic studies support a pathway initiated by LMCT-induced homolysis of iron­(III)-carboxylate complexes to generate alkyl radicals, followed by decarboxylation and RLT-mediated azide transfer from an iron-azide species. The resulting iron­(II) species is reoxidized by nitrate-derived nitrogen oxides, enabling catalytic turnover. Radical trapping experiments, rearrangement probes, kinetic analysis, and UV–vis spectroscopy indicate that radical generation via LMCT is rate-limiting step, while RLT occurs rapidly once the alkyl radical is formed.

**Scheme 16:**
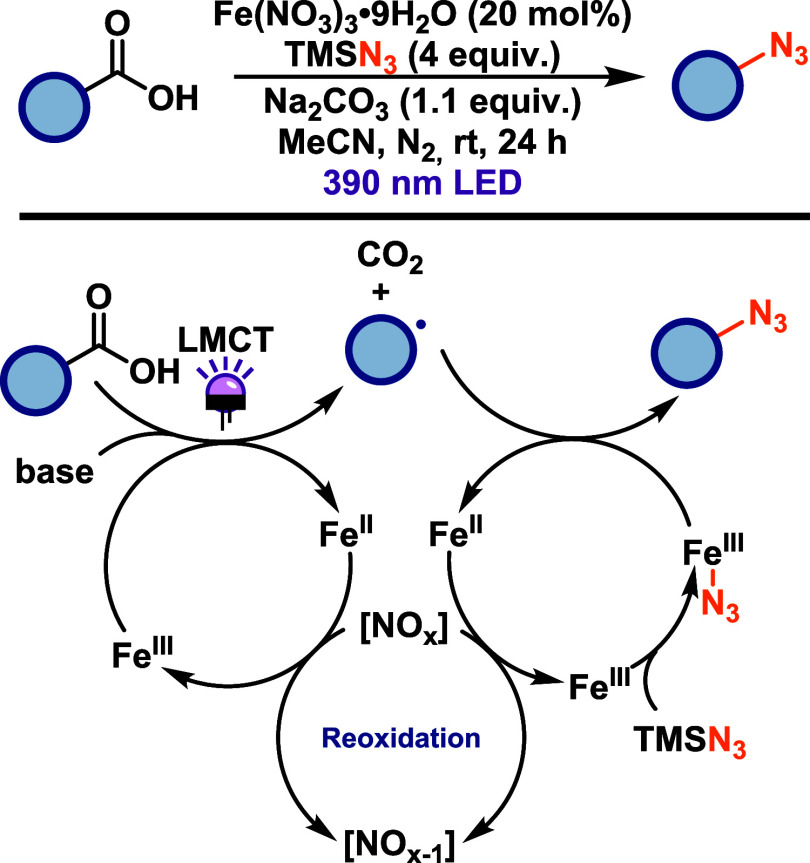
Iron-Catalyzed Azidation of Carboxylic Acids

König and collaborators^37^ developed a blue light-mediated (455 nm), cerium-catalyzed hydrazination of unactivated carboxylic acids using di*tert*-butyl azodicarboxylate (DBAD) as the hydrazine source (Scheme 17). This strategy employs an LMCT activation pathway, enabling radical formation directly from Ce­(III)-carboxylate complexes under mild conditions. The transformation exhibits a broad substrate scope, including primary, secondary, and tertiary aliphatic carboxylic acids, as well as benzylic and amino acid derivatives. Notably, the method exhibits good functional group tolerance, enabling the successful functionalization of carboxylic acids bearing alkenes, alkynes, heterocycles and drug-like scaffolds. Moderate yields were observed for some benzylic acids, possibly due to limited radical stabilization or competing side reactions. The proposed mechanism involves photoinduced LMCT from a Ce­(III)-carboxylate complex to generate a carboxyl radical, which undergoes rapid decarboxylation to form an alkyl radical. This intermediate is trapped by DBAD, forming a nitrogen-centered radical that proceeds through a single electron transfer and protonation sequence to afford the hydrazine product. A base facilitates substrate coordination and is regenerated during the protonation step. The regeneration of Ce­(III) is proposed to occur via oxidation by either the *N*-centered radical or the excited-state DBAD species.

**Scheme 17:**
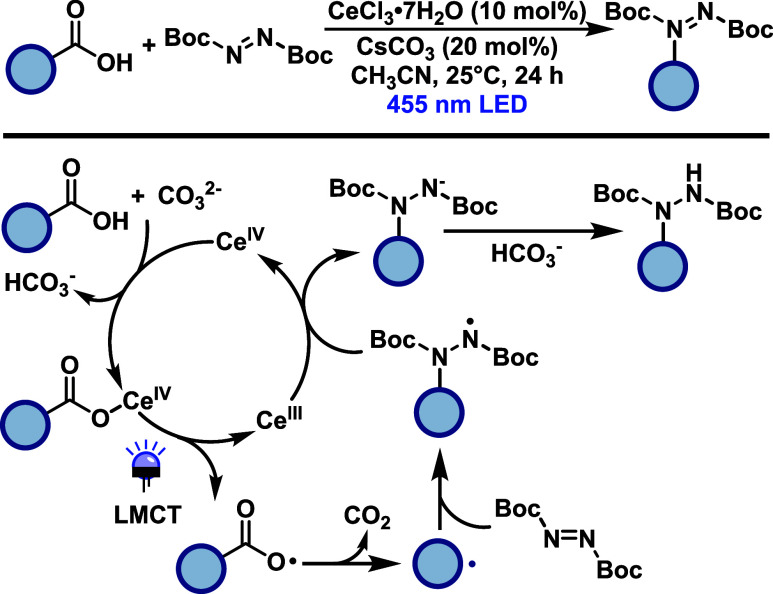
Cerium-Catalyzed Hydrazination of Carboxylic Acids

In 2022, Ritter and co-workers developed a sulfoximination of benzoic acids via LMCT using stoichiometric Cu­(II) under purple light (390 nm).^38^ This transformation enables direct C–N bond formation between aryl carboxylates and NH-sulfoximines (Scheme 18). Benzoic acid derivatives bearing electron-withdrawing substituents, including heteroaromatic carboxylic acids, generally afforded good yields, whereas electron-donating groups were less effective. The reaction tolerates a range of functional groups such as halides, ketones, nitriles, and sulfonamides. The scope of NH-sulfoximines was explored using an optimal benzoic acid partner, yielding moderate efficiency across both alkyl- and aryl-substituted variants, regardless of the electronic nature of the substituents on the sulfur atom. Notably, the reaction is inhibited by the presence of oxidizable functional groups such as amines. Mechanistically, the carboxylate substrate first coordinates to Cu­(II), forming a Cu­(II)-carboxylate complex that undergoes LMCT to generate an aryl carboxyl radical. This intermediate rapidly extrudes CO_2_ to form aryl radical, which is intercepted along with sulfoximine by Cu­(II), forming an aryl-Cu­(III) species. Subsequent reductive elimination furnishes the sulfoximination product.

**Scheme 18:**
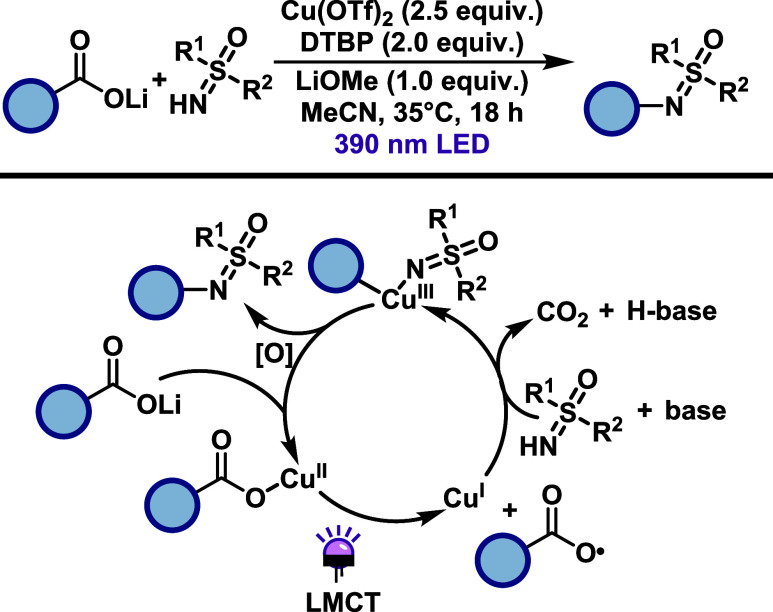
Cu-Mediated Sulfoximination of Benzoic Acids

Jin and co-workers^39^ developed a ligand-accelerated iron photocatalytic system for C–N bond formation using azodicarboxylates as coupling partners (Scheme 19). The methodology employs Fe_2_(SO_4_)_3_ in combination with di­(2-picolyl)­amine and operates under visible light irradiation (427 nm) without the need for external oxidants, enabling a redox-neutral process. In the C–N coupling reaction, dialkyl azodicarboxylates react with a broad range of carboxylic acids to afford hydrazine derivatives. The protocol accommodates α-aryl acetic acids, α-heteroatom-substituted acids, and unactivated aliphatic acids, generating the corresponding primary, secondary, and tertiary radicals under the reaction conditions. The transformation proceeds via the formation of a photoactive Fe­(III)-carboxylate complex. Upon visible light excitation, LMCT induces homolysis to generate a carboxyl radical, which forms an alkyl radical. This radical then engages a coupling reaction with the azodicarboxylate to form the C–N bond. Ligand coordination plays a critical role, as it modulates the absorption profile and redox properties of the iron complex, enabling efficient light-induced reactivity.

**Scheme 19:**
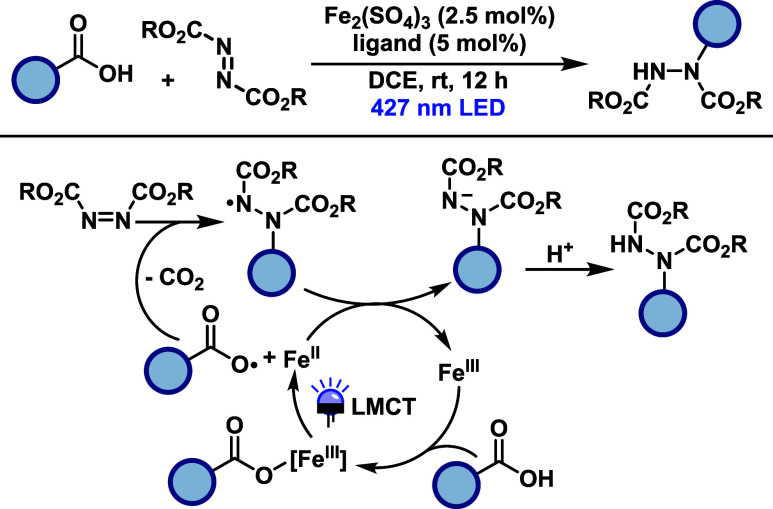
Iron-Catalyzed Hydrazination of Carboxylic Acids

In 2021, Zheng and Wang^40^ developed a metal-free heterogeneous photocatalytic system for C­(sp^3^)-N and C­(sp^3^)-C­(sp^2^) bond formation using boron carbonitride (BCN) as the photocatalyst (Scheme 20). The reaction proceeds under visible light irradiation (420 nm) without the need for transition metals, strong oxidants, or bases, enabling the direct functionalization of carboxylic acids with either N–H or C–H nucleophiles under ambient conditions. The transformation accommodates sterically diverse benzylic acids; however, only highly electron-rich substrates afforded the desired products in moderate to good yields for both C–N and C–C coupling. In the C–N coupling, various azoles act as effective N–H nucleophiles, with electron-donating substituents generally enhancing reactivity. The system exhibits good recyclability over multiple cycles with minimal loss of activity. Mechanistically, the process involves photoexcitation of the BCN semiconductor generating electron–hole pairs. The valence band hole oxidizes the carboxylic acid, triggering decarboxylation and formation of an alkyl radical. In the C–N coupling pathway, this radical is intercepted by an azole to form a new radical intermediate, which undergoes single-electron oxidation followed by deprotonation the final product (path A). The addition of 4-hydroxy-TEMPO facilitates hydrogen atom abstraction and propagates the radical process (path B), while the solvent 1,2-dichloroethane (DCE) functions both as the reaction medium and as an electron acceptor.

**Scheme 20:**
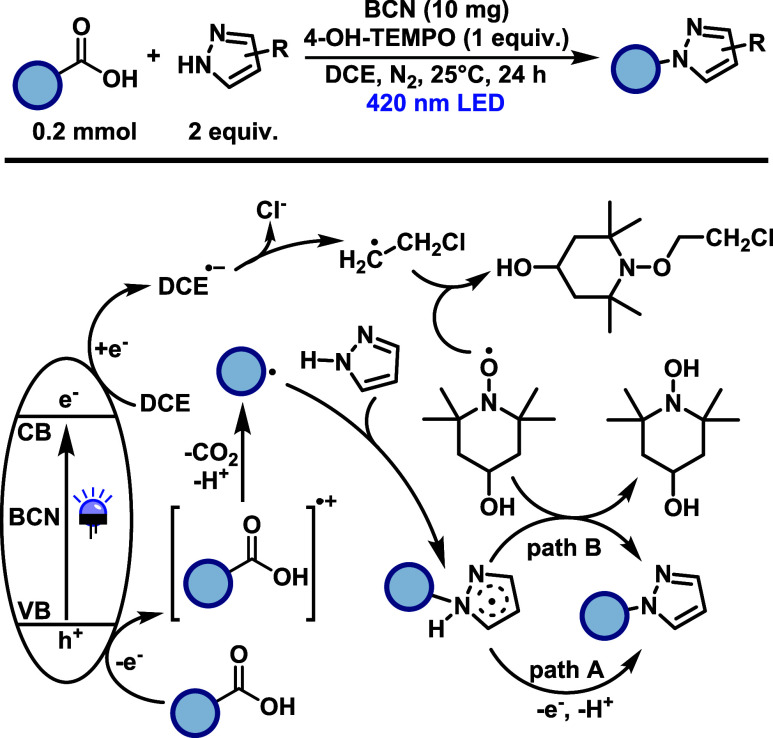
Boron Carbonitride Heterogeneous Photocatalysis for C­(sp^3^)-N Bond Formation

Yoon and co-workers^41^ developed a copper-mediated cross-nucleophile coupling protocol for C–N, C–O, and C–C bond formation from carboxylic acids, without the need for external photosensitizers, oxidants, or high-cost metals (Scheme 21). The reaction is compatible with a wide range of nucleophiles, including sulfonamides, carbamates, amides, alcohols, and electron-rich (hetero)­arenes. Electron-rich arylacetic acids consistently deliver the best results, both sterically hindered substrates and functionalized drug-like acids are well tolerated. Functional groups such as halides, esters, ketones, and heterocycles are tolerated under the reaction conditions. The mechanism begins with LMCT excitation of the Cu­(II)-carboxylate complex, initiating homolysis to generate a carboxyl radical, which then undergoes decarboxylation to form a radical. This radical is oxidized to a carbocation or high-valent copper species, which is subsequently intercepted by the nucleophile to form the desired C–N, C–O, or C–C bond. UV–vis spectroscopy and stoichiometric studies support the involvement of photoactive copper species and the formation of higher-order aggregates under conditions of excess carboxylate was found to diminish reactivity.

**Scheme 21:**
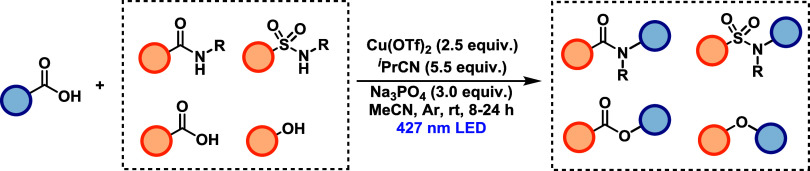
Copper-Mediated Cross-Nucleophile Coupling Protocol for C–N and C–O Bond Formation

Zhu and co-workers^42^ developed an iron-catalyzed, visible-light-driven decarboxylative C–N coupling between benzylic carboxylic acids and nitroarenes, enabling the synthesis of tertiary anilines under redox-neutral conditions (Scheme 22). The protocol enables the formation of two C–N bonds in a single reaction using a porphyrin-iron complex, which prevents overalkylation. Although the scope is currently limited to benzylic acids, the method allows for the selective coupling of two structurally distinct carboxylic acids to afford the corresponding aniline derivative. The proposed mechanism involves coordination of the iron catalyst to the carboxylic acid, forming a Fe­(III)-carboxylate complex that undergoes photoinduced LMCT to generate a carboxyl radical. Subsequent decarboxylation furnishes a benzylic radical, which attacks a nitrosoarene intermediate generated *in situ* via partial reduction of the nitroarene. This leads to a transient *N*-centered radical, which undergoes C–N bond formation via an S_H_2 (bimolecular homolytic substitution) mechanism. The redox-neutral nature of the reaction is maintained through the internal cycling of the iron oxidation states.

**Scheme 22:**
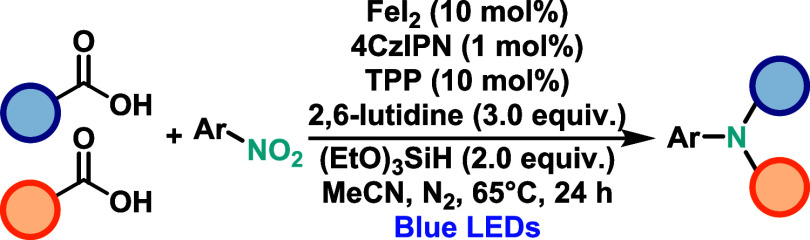
Iron-Catalyzed Decarboxylative C–N Coupling between Benzylic Carboxylic Acids and Nitroarenes

Yu and co-workers^43^ developed an iron-catalyzed C–N coupling of alkyl carboxylic acids with sodium nitrite as a nitric oxide (NO) source to access oximes (Scheme 23). The protocol uses Fe­(NO_3_)_3_·9H_2_O as the catalyst and proceeds under mild conditions without the need for photosensitizers, external oxidants, or reductants. This method enables *in situ* NO generation from NaNO_2_ and offers operational simplicity. The scope includes a variety of benzylic acids bearing electron-donating and electron-withdrawing groups, halogens, and heteroaryl motifs, all affording good to excellent yields. While primary and secondary benzylic acids perform well, nonbenzylic acids and complex frameworks such as dehydrocholic acid led to reduced yields. The proposed mechanism begins with ligand exchange between Fe­(III), the carboxylic acid and NO_2_^–^, forming a Fe­(III)-carboxylate-nitrite complex. Upon photoexcitation, LMCT generates a carboxyl radical and a Fe­(II)-NO_2_ intermediate. The carboxyl radical undergoes decarboxylation to form an alkyl radical, while the Fe­(II)-NO_2_ species is protonated and dehydrates to form Fe-NO complex. The alkyl radical subsequently couples with the released NO to form a nitroso intermediate, which rearranges to the final oxime product.

**Scheme 23:**
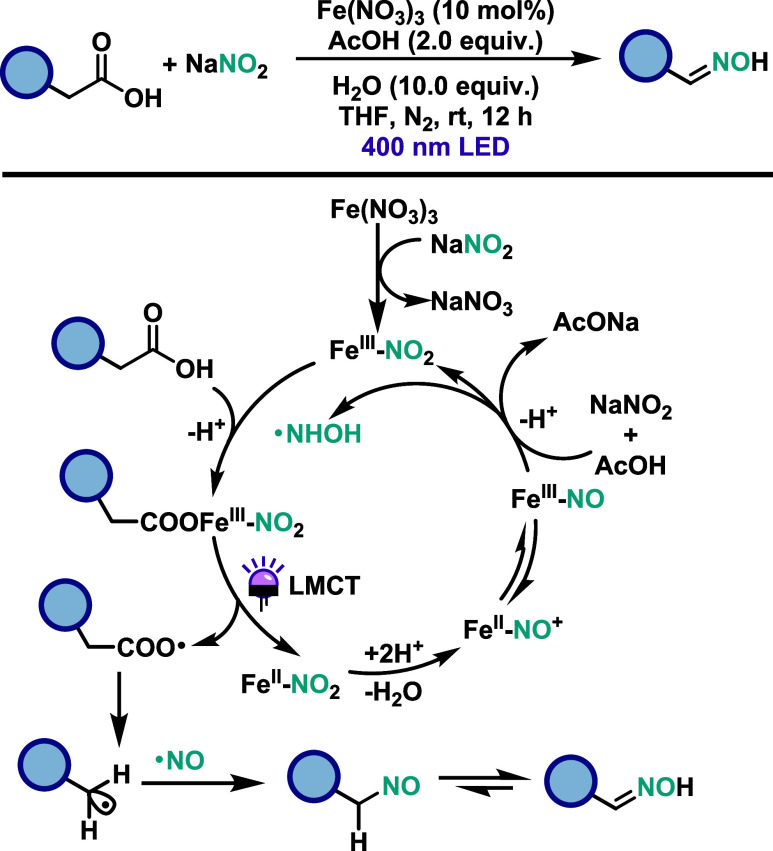
Iron-Catalyzed C–N Coupling of Alkyl Carboxylic Acids with Sodium Nitrite to Access Oximes

Murakami and co-workers^44^ developed a method for the functionalization of arylacetic acids to enable C­(sp^3^)-N, C­(sp^3^)-O, and C­(sp^3^)-Cl bond formation using unactivated nitrogen, oxygen, and chloride nucleophiles (Scheme 24). The transformation is mediated by Ru­(bpy)_3_Cl_2_ and a hypervalent iodine oxidant (IBB) and proceeds under blue light irradiation without requiring prefunctionalization of either coupling partner. The method tolerates a range of arylacetic acids bearing electron-donating or halogen substituents, including naphthalene, thiophene, and indole derivatives. Electron-deficient acids exhibit lower reactivity under these conditions. Imide nucleophiles couple efficiently regardless of aryl or alkyl substitution. Additional electrophilic nucleophiles such as trifluoroacetic acid, phosphonic acid, and fluoroalcohols also participate, affording C–O coupled products. Chlorination is achieved using tetrabutylammonium chloride, followed by S_N_2 reactions on the resulting benzyl chlorides. The method is applicable to gram-scale synthesis and late-stage functionalization of pharmaceuticals containing arylacetic acid motifs. The proposed mechanism begins with the formation of an iodine­(III) carboxylate intermediate from the reaction between the acid and IBB. Upon visible-light excitation, Ru­(II)* reduces the iodine­(III) species, initiating the decarboxylation to generate a benzyl radical. Simultaneously, Ru­(III) oxidizes the nitrogen or oxygen nucleophile to its corresponding radical, which couples with the benzyl radical to form the desired product. The ring-opening observed with a cyclopropyl-substituted acid supports the involvement of radical intermediates. Alternative ionic pathways are considered plausible, depending on the nature of the nucleophile.

**Scheme 24:**
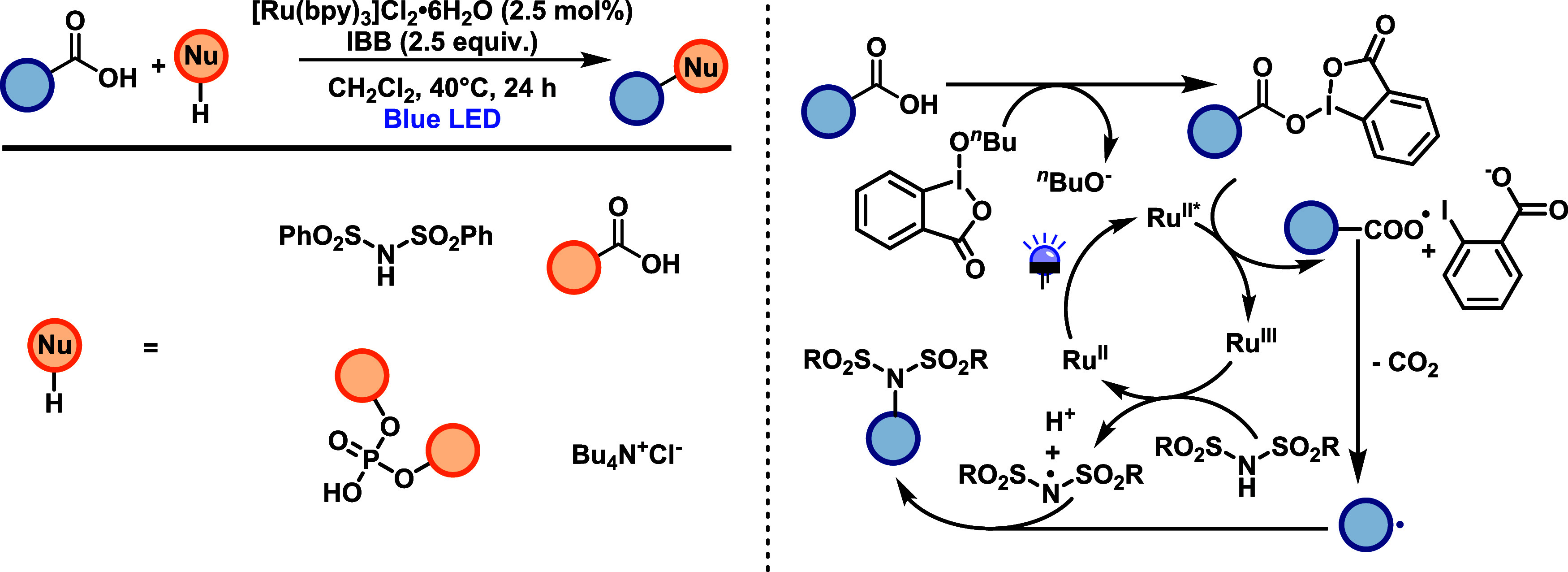
Functionalization of Arylacetic Acids to Enable C­(sp^3^)-N, C­(sp^3^)-O, and C­(sp^3^)-Cl Bond Formation

MacMillan and co-workers developed a dual catalytic platform for C­(sp^3^)-N bond formation, combining iridium and copper catalysis (Scheme 25). The method enables direct construction of C–N bonds from alkyl carboxylic acids and a broad array of nitrogen nucleophiles under mild conditions.^45^ The transformation exhibits moderate functional group tolerance and is compatible with primary, secondary, and tertiary alkyl carboxylic acids. However, unlike decarboxylation protocols involving LMCT, this methodology is not applicable to benzylic acids. The scope of nitrogen nucleophiles includes azoles, sulfonamides, anilines, amides, and various heterocycles, demonstrating high compatibility across diverse substitution patterns. The strategy is suitable for late-stage functionalization and gram-scale synthesis. In the proposed mechanism, a photoexcited Ir­(III) photocatalyst oxidizes an *in situ*-generated iodonium carboxylate to give a carboxyl radical. Decarboxylation yields an alkyl radical, which is intercepted by a Cu­(II)-nucleophile complex to form a Cu­(III) intermediate. Subsequent reductive elimination furnishes the C–N bond and regenerates Cu­(I) species. The photoredox cycle is completed by reduction of Ir­(IV) by Cu­(I), thus closing the dual catalytic cycle.

**Scheme 25:**
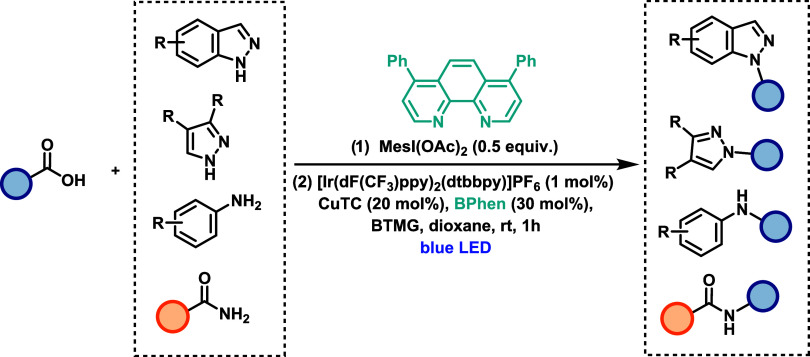
Iridium-Catalyzed Decarboxylative C­(sp^3^)-N Coupling

## Carbon-Halogen Bonds

Alkyl and aryl halides are fundamental building blocks in synthetic chemistry. Accordingly, the development of methodologies for constructing carbon–halogen (C–X, where X = halogen) bonds remains of significant interest to chemists.^46^ Decarboxylative halogenation reactions have their origins in the pioneering work of Borodine, Hunsdiecker, Kochi, and Barton.^47–51^ Traditional methods typically involve the use of toxic and environmentally hazardous metals such as Ag, Hg, Pb, Ti, or Tl, as well as harsh reaction conditions, and hazardous halogen sources like Br_2_ or Cl_2_.^52^ In recent years, however, photochemical methodologies have emerged as milder and safer alternative for achieving C–X bond formation via decarboxylative halogenation.

In this context, Glorius and co-workers^46^ reported the first catalytic Hunsdiecker-type bromination of alkyl carboxylic acids, along with its extension to chlorination and iodination (Scheme 26). The transformation was achieved using the photocatalyst [Ir­(dF­(CF_3_)­ppy)_2_(dtbbpy)]­PF_6_, Cs_2_CO_3_ as the base, and diethyl bromomalonate as the bromine source. A blue LED lamp (455 nm) served as the light source, and the reaction proceeded within just 4 h, affording moderate to good yields across a range of substrates, including primary, secondary, and tertiary alkyl carboxylic acids. Attempts to perform chlorination using diethyl chloromalonate were unsuccessful, likely due to the higher bond dissociation energy of the C–Cl bond. Similarly, the use of ethyl iodoacetate as an iodine source predominantly resulted in an S_N_2 product, attributed to its high electrophilicity. Subsequent optimization revealed that *N*-chlorosuccinimide (NCS) and its iodine analogue, *N*-iodosuccinimide (NIS), are effective halogenating agents, affording the corresponding alkyl halides in moderate to good yields. However, these reactions required extended irradiation times of up to 14 h. The proposed mechanism begins with photoexcitation of the Ir­(III) photocatalyst via a metal-to-ligand charge transfer (MLCT) process.^3^ The excited state photocatalyst functions as a strong oxidant, promoting a single-electron oxidation of the alkyl carboxylate. Decarboxylation of the resulting carboxylate radical generates the corresponding alkyl radical, which undergoes halogen atom transfer from the halogen source to afford the final product. Electron transfer from the Ir­(II) species to the resulting malonyl radical or succinimide radical regenerates the photocatalyst (Scheme 26).

**Scheme 26:**
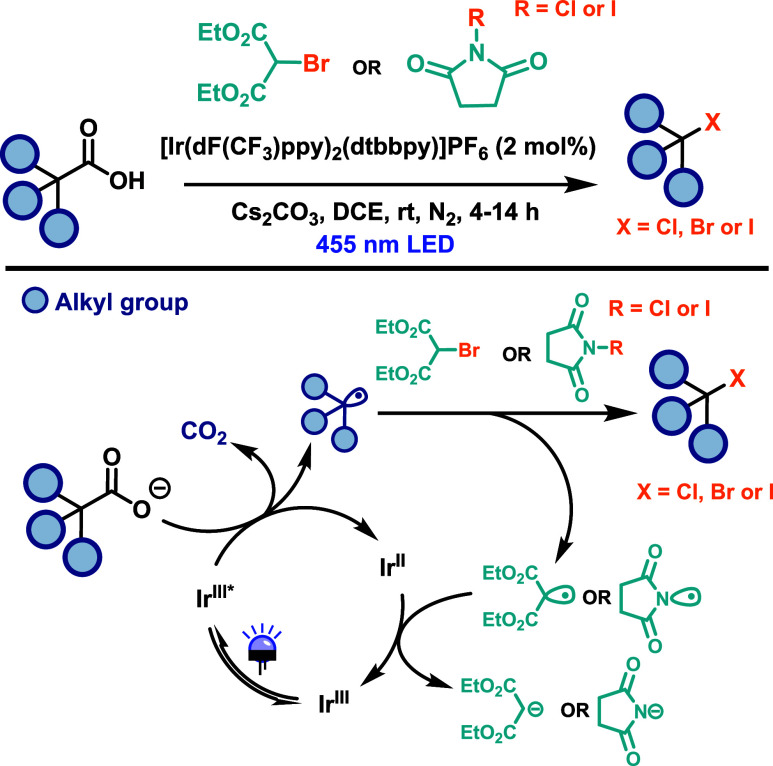
Iridium-Catalyzed Decarboxylative Halogenation of Carboxylic Acids

A similar protocol was developed by Fu and co-workers,^53^ targeting on the synthesis of aryl and heteroaryl iodides (Scheme 27), giving their relevance in transition metal-catalyzed cross-coupling chemistry. This method employed the same photocatalyst, base, solvent, and LED wavelength as those used by Glorius and co-workers. ([Ir­(dF­(CF_3_)­ppy)_2_(dtbbpy)]­PF_6_, Cs_2_CO_3_, DCE, and 455 nm, respectively). *N*-Iodosuccinimide (3 equiv) was used as the iodine source, along with the addition of a catalytic amount of I_2_. However, the reaction required heating at 50 °C for 24–36 h to proceed efficiently. Although limited to aromatic carboxylic acids, the protocol demonstrated good functional group tolerance and a broad substrate scope.

**Scheme 27:**
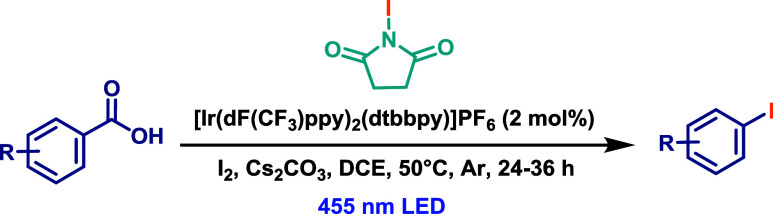
Decarboxylative Iodination of Aromatic Carboxylic Acids

Although Ir­(III)-based photocatalysis is highly effective and offers several advantages due to the favorable photophysical properties of iridium complexes, the high cost and limited availability of iridium remain major obstacles to large-scale applications.^54^ As a result, the search for alternative photocatalysts has shifted chemists’ attention toward more abundant 3d transition metals capable of undergoing LMCT processes under visible light irradiation. The application of LMCT chemistry to promote C–X bond formation was explored by the MacMillan group as a unified strategy for the decarboxylative halogenation of aryl carboxylic acids (Scheme 28).^55^ In this work, the Cu­(I) complex [Cu­(MeCN)_4_]­BF_4_ served as the copper source and was reacted with the oxidant 1-fluoro-2,4,6-trimethylpyridinium tetrafluoroborate (NFTPT) to generate a Cu­(II) photoactive carboxylate complex. Irradiation was carried out using a 365 nm LED lamp. For the bromination reaction, 20 mol % of the catalyst and 1,3-dibromo-5,5-dimethylhydantoin (DBDMH) as the bromine source were employed. Upon photoexcitation, the Cu­(II) carboxylate complex undergoes an LMCT process, generating a carboxylate radical that rapidly decarboxylates to form an aryl radical. This aryl radical then engages in an atom-transfer reaction with DBDMH to afford the brominated product. The LMCT cycle reduces Cu­(II) back to Cu­(I), which is subsequently reoxidized by NFTPT to regenerate the active Cu­(II) species. The reaction proceeds at room temperature and completes within 6 h. The iodination reaction followed a similar protocol, with *N*-iodosuccinimide (NIS) serving as the iodine source. In contrast, chlorination required a higher loading (up to 1 equiv of Cu (I)), ZnCl_2_ as the chlorine source, and extended reaction times (up to 12 h). In this case, the mechanism diverged after aryl radical formation, involving coordination of the aryl radical and chloride anion to Cu­(II) to form a high-valent aryl–Cu­(III) intermediate. Reductive elimination from this intermediate yields the chlorinated product and regenerates the Cu­(I) species (Scheme 28). Similarly, fluorination required 3 equiv of [Cu­(MeCN)_4_]­BF_4_, with NFTPT acting both as the oxidant and fluorine source. This transformation also proceeded via a Cu­(III)-mediated pathway, with reaction times up to 24 h. Overall, the halogenated aryl products were obtained in moderate to good yields under mild conditions, with broad functional group tolerance. The authors further demonstrated the versatility of the protocol by performing late-stage functionalization of biologically active molecules, as well as one-pot *in situ* S_N_Ar reactions between the aryl fluorides and various nucleophiles, yielding C–N, C–O, and C–S bond-forming products. These results highlight the broad applicability and robustness of the method.

**Scheme 28:**
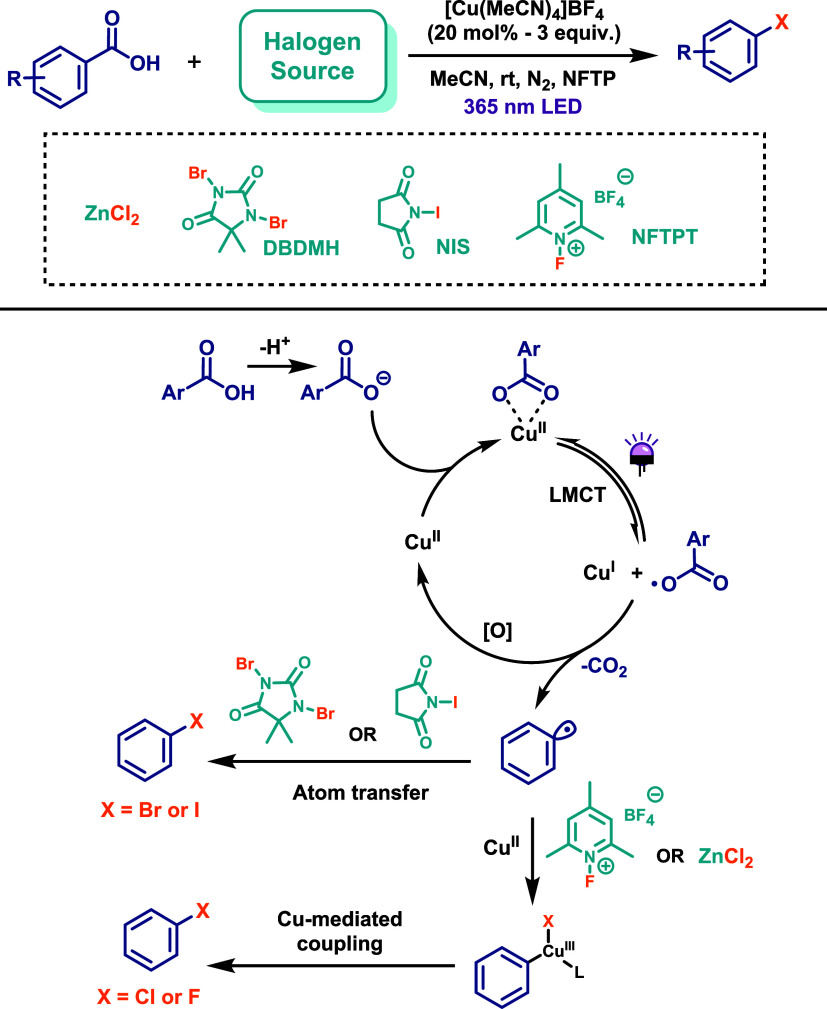
Unified Protocol to Achieve Decarboxylative Halogenation of Aryl Carboxylic Acids via Cu­(II) Photocatalysis

The LMCT strategy also enables more environmentally friendly methodologies by allowing the use of milder reaction conditions to achieve the desired transformations. For example, Jin and co-workers^52^ developed a decarboxylative halogenation of aliphatic carboxylic acids via Ce­(IV)-mediated LMCT photocatalysis, taking advantage of the earth abundance and low environmental impact of cerium salts. The transformations were carried out using 10 mol % CeCl_3_ as the photocatalyst, 30 mol % *t*-BuONa as the base, and *N*-bromosuccinimide (NBS), *N*-iodosuccinimide (NIS), or trichloroisocyanuric acid (TCCA) as the bromine, iodine, and chlorine sources, respectively, under blue LED irradiation (Scheme 29). Notably, one of the most innovative features of this protocol was the use of water as the reaction solvent under ambient air, significantly enhancing its sustainability. The reaction proceeds via a typical LMCT catalytic cycle, in which molecular oxygen present in the reaction medium plays a crucial role in reoxidizing Ce­(III) to Ce­(IV), thereby closing the photocatalytic cycle. This protocol proved highly effective, exhibiting good functional group tolerance and broad substrate scope, including aliphatic carboxylic acids derived from pharmaceutical compounds and natural products.

**Scheme 29:**
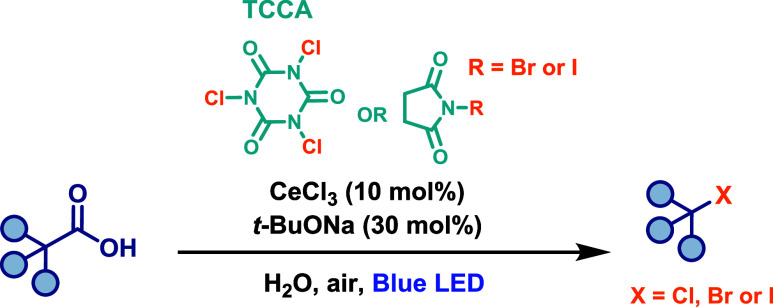
Cerium-Catalyzed Decarboxylative Halogenation of Aliphatic Carboxylic Acids

More recently, a specific protocol for the decarboxylative bromination of aryl carboxylic acids was reported by Deng and Jin.^56^ This Iron-catalyzed approach employs FeBr_2_ as a precatalyst and sodium bromate (NaBrO_3_) as both the bromine source and oxidant, facilitating the conversion of Fe­(II) into the photoactive Fe­(III) species (Scheme 30). The reaction was conducted in MeCN under the irradiation with 440 nm LED lamps for 10 h. However, this methodology requires large amounts of the oxidants NaBrO_3_ (2.5 equiv) and trifluoroacetic (TFA, 7.5 equiv), both to generate the LMCT-active Fe­(III) complexes from the precatalyst and to complete the catalytic cycle.

**Scheme 30:**
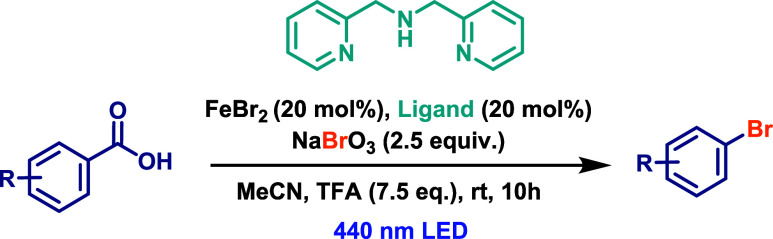
Iron-Catalyzed Decarboxylative Bromination of Aryl Carboxylic Acids

Among halogenated organic compounds, fluorinated molecules play a particularly important role in industry due to their broad applications in pharmaceuticals (owing to their biological activity), agrochemicals, and polymers.^57^ However, traditional fluorination methods suffer from several drawbacks, particularly in terms of safety and environmental impact, which has increased the demand for safer, more practical, and cost-effective alternatives.^58^ In this context, the decarboxylative fluorination of carboxylic acids has emerged as a promising strategy for synthesis of organofluorine compounds. Hu and colleagues^59^ developed a simple and effective protocol for the decarboxylative fluorination of aliphatic carboxylic acids using Fe­(III)-mediated LMCT catalysis. The transformation employed Fe­(OAc)_2_ (10 mol %) as the catalyst, with an additional 20 mol % of a ligand, 2,6-lutidine (1.8 equiv) as the base. Selectfluor (2.1 equiv) serve as both fluorine source and oxidant, and a 1:1 mixture of MeCN/H_2_O was used as the solvent (Scheme 31). The reaction was carried out under blue LED irradiation (455 nm) for only 2 h. The desired fluorinated products were obtained in moderate to good yields, displaying broad functional group tolerance and high selectivity. However, the method was found to be incompatible with aryl carboxylic acids. The proposed reaction mechanism begins with the oxidation of Fe­(II) to photoactive Fe­(III), which forms an iron carboxylate complex that absorbs light via an LMCT process. The resulting alkyl radical, generated after decarboxylation, undergoes an atom-transfer reaction with Selectfluor to yield the fluorinated product (Scheme 31). This method offers several advantages, particularly the use of iron salts as catalysts. As iron is an abundant, low-cost, and environmentally benign metal, this protocol has the potential to serve as a practical and economic alternative for accessing fluorinated organic compounds on a large-scale. Furthermore, the authors demonstrated its utility in the late-stage functionalization of complex molecules, including pharmaceuticals and natural products.

**Scheme 31:**
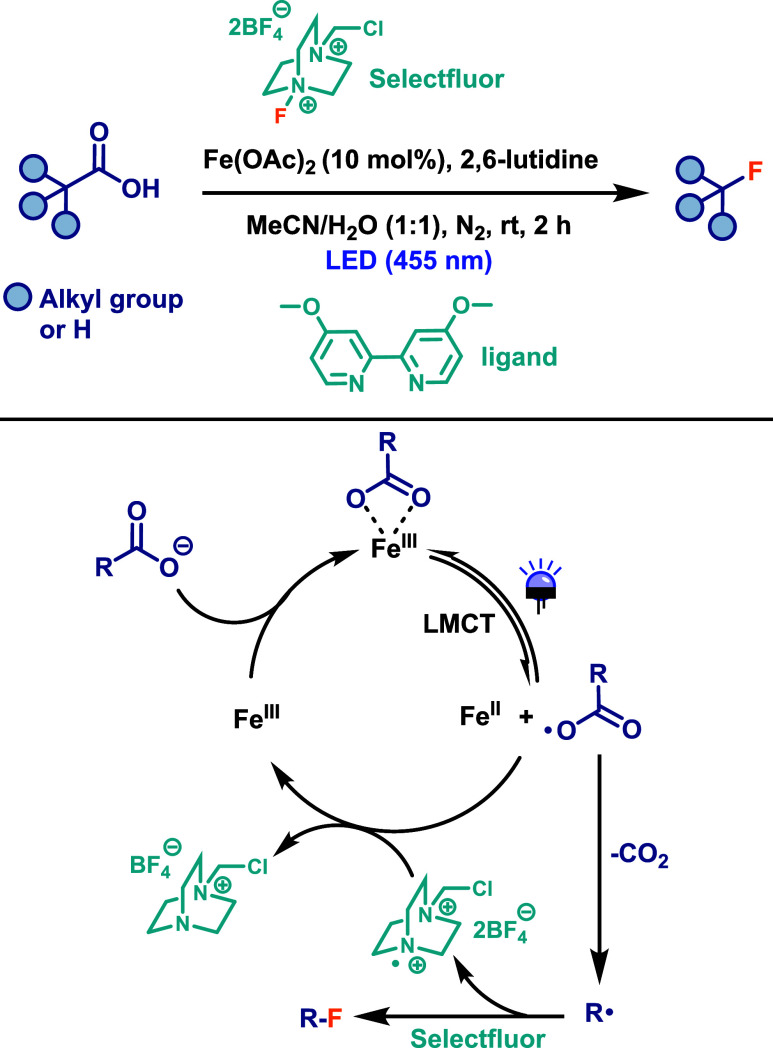
Iron-Catalyzed Decarboxylative Fluorination of Aliphatic Carboxylic Acids

The synthesis of aryl fluorides via direct radical decarboxylation remains challenging, as aryl radicals are less nucleophilic than their aliphatic counterparts. To address this limitation, Ritter and co-workers developed a novel strategy.^60^ This protocol involves the reaction of aryl carboxylic acids with tetrabutylammonium tetra­(*t*-butyl alcohol)-coordinated fluoride (TBAF·(*t*-BuOH)_4_, 2.5 equiv) in the presence of stoichiometric amounts of copper salts such as Cu­(OTf)_2_ or Cu­(MeCN)_4_BF_4_, under purple LED irradiation at 35 °C for 24 h (Scheme 32). The reaction mechanism begins with the photoinduced generation of aryl radicals via a Cu­(II) LMCT process. The resulting aryl radical and fluoride anion then coordinate with copper to form a hypervalent aryl–Cu­(III) intermediate, which undergoes reductive elimination to afford the aryl fluoride product. As the reaction employs stoichiometric amounts of copper, reoxidation of Cu­(I) to Cu­(II) is not required. This method provides aryl fluorides in good yields and demonstrates broad functional group compatibility. However, it was found to be less effective with heteroaromatic acids and benzoic acids bearing strongly coordinating or easily oxidizable groups, such as amines.

**Scheme 32:**
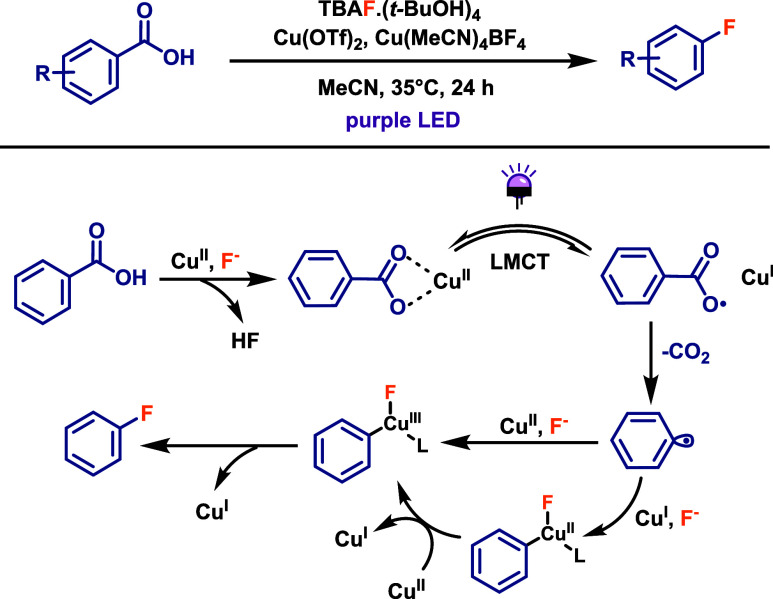
Cu-Promoted Decarboxylative Fluorination of Aryl Carboxylic Acids

## Carbon-Sulfur Bonds

Sulfur-containing organic molecules are widely used across various fields, including medicinal chemistry and materials science. However, due to the diverse oxidation states that sulfur can adopt in organic compounds, the selective formation of carbon–sulfur bonds with a specific sulfur oxidation state remains a significant synthetic challenge. In this context, Larionov and co-workers reported a strategy for accessing sulfoxides via the decarboxylative sulfinylation of carboxylic acids.^61^ Sulfoxides, which can be considered as intermediates between sulfides and sulfones in terms of oxidation state, are crucial scaffolds in both pharmaceuticals and agrochemicals. The transformation involves the reaction of aliphatic carboxylic acids with sodium aryl sulfinates in the presence of acridine (10 mol %) as a photocatalyst and *p*-bromobenzoyl chloride (PBC) under 400 nm LED irradiation for 12 h (Scheme 33). This method affords the desired sulfoxides in moderate to good yields and exhibits broad functional group tolerance. Notably, the reaction was successfully applied to the late-stage functionalization of natural products and pharmaceutical compounds. The proposed mechanism (Scheme 33) begins with the coordination of the carboxylic acid to the acridine photocatalyst in its ground state. Upon light absorption, a PCET occurs, followed by decarboxylation to form an alkyl radical. Simultaneously, the sulfinate anion is activated by PBC to form a mixed anhydride, which reacts with a second equivalent of sulfinate to generate a sulfinyl sulfone intermediate in situ. The alkyl radical then couples with the sulfinyl sulfone to furnish the desired sulfoxide. This protocol offers a selective, operationally simple, metal-free, and one-step synthesis of sulfoxides from carboxylic acids under mild conditions. It represents the first reported example of sulfoxide formation via radical substitution of intermediate sulfinyl sulfones.

**Scheme 33:**
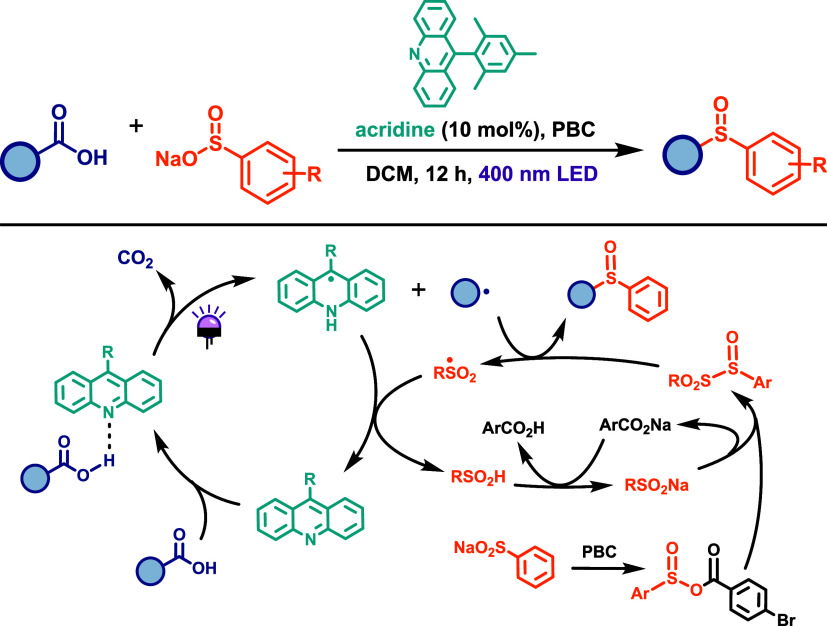
Metal-Free Synthesis of Sulfoxides by Decarboxylative Sulfinylation of Carboxylic Acids

Larionov’s group also demonstrated the feasibility of acridine-catalyzed decarboxylation to access sulfones via a dual-catalytic mechanism.^62^ In this study, they developed a three-component decarboxysulfonylative cross-coupling of carboxylic acids and aryl halides in the presence of a sulfur dioxide (SO_2_) source, employing a dual acridine/copper catalytic system. The reaction was carried out under 400 nm LED irradiation for 14 h, using 10 mol % of the acridine photocatalyst, 10 mol % of CuOTf, and either BABSO or K_2_S_2_O_5_ as the SO_2_ source (Scheme 34). Despite its efficacy, the transformation required elevated temperatures (90–100 °C) to proceed efficiently. The method exhibited good functional group tolerance but was limited to aliphatic carboxylic acids and aryl halides (bromides and iodides). The proposed reaction mechanism begins with alkyl radical generation via acridine-catalyzed photodecarboxylation (similar to the mechanism on Scheme 33). This radical is subsequently trapped by SO_2_ to generate a sulfonyl radical, which undergoes copper-catalyzed cross-coupling with the aryl halide to afford the corresponding sulfone product. This protocol represents the first direct, one-step conversion of carboxylic acids and (hetero)­aryl halides into alkyl (hetero)­aryl sulfones.

**Scheme 34:**
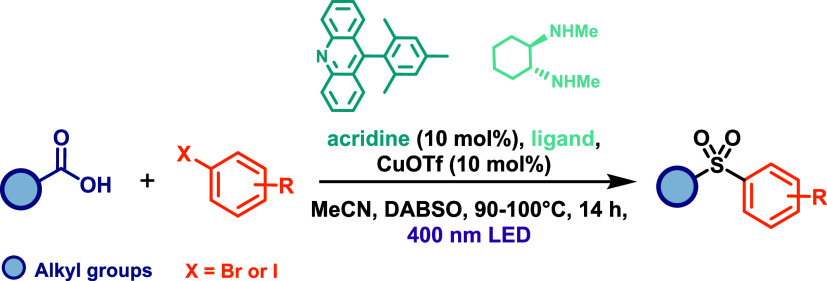
Three-Component Decarboxysulfonylative Cross-Coupling of Carboxylic Acids and Aryl Halides

Similarly, an analogous protocol was developed by the same group for the synthesis of sulfonamides and their derivatives.^63^ Initially, the methodology was applied to the synthesis of *N*-alkyl sulfonamides via the reaction of aliphatic carboxylic acids with *O*-benzoylhydroxylamines, using 10 mol % acridine photocatalyst, 10 mol % CuF_2_, and DABSO as the sulfur dioxide source, under 400 nm LED irradiation for 12 h. The protocol was further extended to the synthesis of *N*-aryl sulfonamides and sulfonyl azides with only minor modifications to the general procedure (Scheme 35). Remarkably, the reaction proceeds efficiently with both electrophilic and nucleophilic nitrogen-centered coupling partners, enabling the concomitant formation of C–S and S–N bonds. This versatility renders the protocol a powerful approach for the efficient synthesis of structurally diverse sulfonamides, thereby expanding access to a broad region of chemical space. Furthermore, the same strategy was later shown to be effective for direct decarboxylative chloro- and fluorosulfonylation, enabling simultaneous formation of C–S and C-X (X = Cl or F) bonds under similar conditions.^64^

**Scheme 35:**
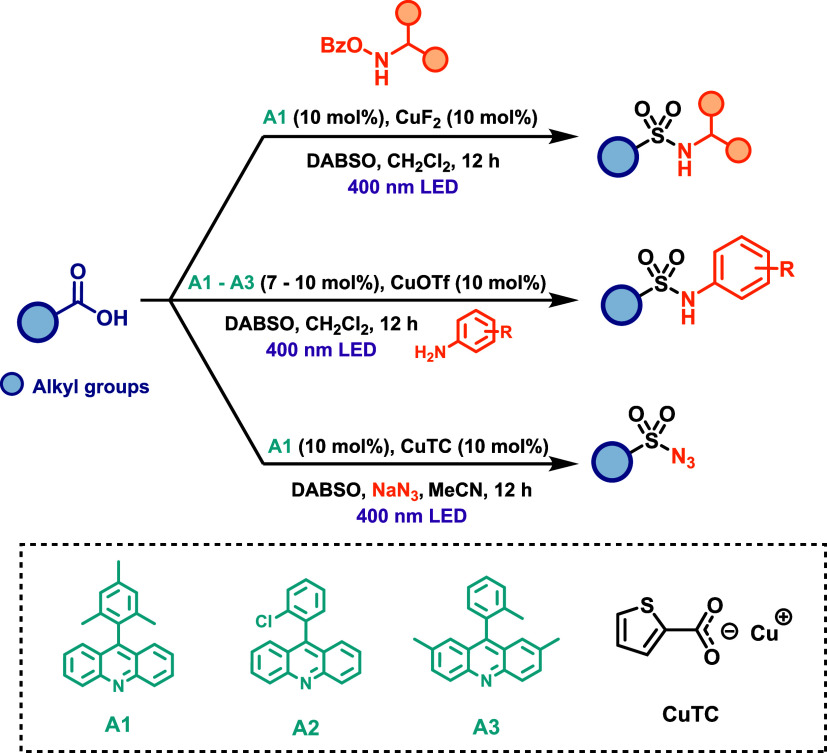
Three-Component Decarboxylative Amidosulfonation of Carboxylic Acids

More recently, the synthesis of organic sulfides has been achieved via an iron-catalyzed decarboxylative thiolation of both aliphatic and aromatic carboxylic acids, employing aryl thiosulfonates as radical coupling partners.^65,66^ These methodologies utilize Fe­(III) salts, particularly Fe­(NO_3_)_3_·9H_2_O, as catalysts in the presence of a base (such as sodium or potassium carbonates) under irradiation with a 390 nm LED lamp (Scheme 36). The reaction proceeds through a classical Fe­(III) LMCT catalytic mechanism. Notably, the protocol is compatible with both aliphatic and aromatic carboxylic acids, affording the corresponding sulfides in moderate to good yields. This is particularly significant given that, under mild conditions, the direct decarboxylation of aromatic acids is approximately a thousand times slower than that of their aliphatic counterparts.^67^ Additionally, the reaction atmosphere plays a critical role in determining chemoselectivity: under a nitrogen atmosphere, thiolation predominates, leading to the formation of sulfides, whereas under air sulfinylation occurs, yielding sulfoxides as the final products (Scheme 36).

**Scheme 36:**
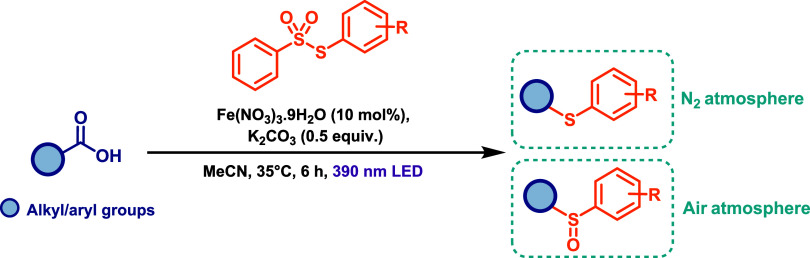
Iron-Catalyzed Decarboxylative Thiolation and Sulfinylation of Carboxylic Acids

## Carbon-Selenium Bonds

Selenylated compounds are widely used across several fields, including medicinal chemistry, organic synthesis, and materials science.^68^ However, the formation of C–Se bonds via radical decarboxylation of carboxylic acids remains relatively underexplored. Zhang and co-workers^69^ reported a transition-metal-free oxidative trifluoromethylselenolation of aliphatic carboxylic acids under visible-light irradiation. This methodology employed [Me_4_N]­[SeCF_3_] as the SeCF_3_ source. Several trifluoromethylselenolation reagents capable of enabling direct C–Se bond formation has been reported, including Hg­(SeCF_3_)_2_, CuSeCF_3_, [(bpy)­CuSeCF_3_]_2_, [Me_4_N]­[SeCF_3_], ClSeCF_3_, and TsSeCF_3_. Among these, [Me_4_N]­[SeCF_3_] is by far the most widely used nucleophilic SeCF_3_ source due to its thermally stability, low cost, ready availability, nonvolatility, ease of handling, and straightforward synthesis.^70^ The desired transformation was achieved using an acridine as a photocatalyst (1 mol %) under blue LED irradiation for 24 h at room temperature, with *N*-fluorobenzenesulfonimide (NFSI) serving as the oxidant (Scheme 37). The proposed reaction mechanism begins with acridine-catalyzed decarboxylation to generate an alkyl radical. This then reacts with ^•^SeCF_3_, which is formed through oxidation of [Me_4_N]­[SeCF_3_] by NFSI, affording the trifluoromethylselenylated product. Alternatively, the alkyl radical may react with F_3_CSeSeCF_3_, whose in situ formation was confirmed by ^19^F NMR analysis of the reaction mixtures. Moreover, the oxidant plays a dual role: it reoxidizes the reduced photocatalyst and its reduced forms can also serve as bases to deprotonate the carboxylic acid, facilitating carboxylate formation and subsequent photoinduced decarboxylation. Nevertheless, the exact mechanism remains to be fully elucidated.

**Scheme 37:**
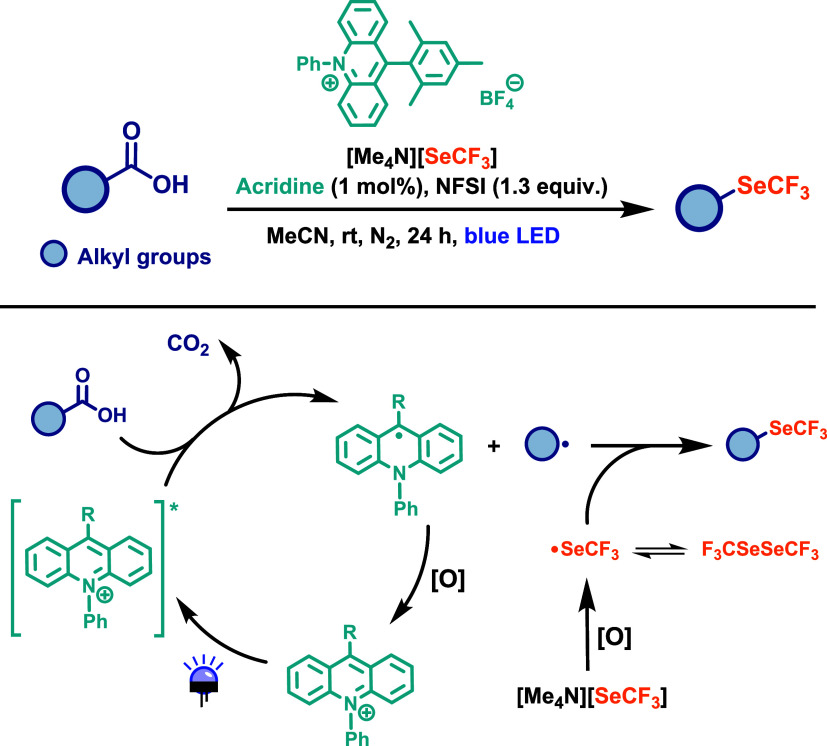
Transition-Metal-Free Oxidative Trifluoromethylselenolation of Aliphatic Carboxylic Acids

C–Se bond formation was also investigated by Liu and co-workers,^71^ who reported a visible-light-induced radical decarboxylative coupling of α-oxo acids with diselenides to produce selenol esters under photocatalyst- and oxidant-free conditions. The reaction was carried out by directly irradiating α-keto acids and diselenides with blue LED light for 48 h under an air atmosphere (Scheme 38). The protocol was found to be effective for both aryl and alkyl diselenides; however, its efficiency was limited to aromatic α-keto acids, with significantly lower yields observed for aliphatic acids. The proposed mechanism starts with photoexcitation of the diselenide which interacts with a molecule of oxygen to generate singlet oxygen (^1^O_2_) via energy transfer (EnT). The singlet oxygen then reacts with the α-keto acid, promoting decarboxylation and forming an acyl radical. A final step of radical coupling with diselenide affords the selenoester product. Alternatively, in the absence of oxygen, the radical decomposition of the α-keto acids under visible light leads to the formation of acyl radicals that subsequently couple with diselenides. Notably, diselenides have been shown to possess excellent radical-trapping ability toward carbon-centered radicals.^72^

**Scheme 38:**
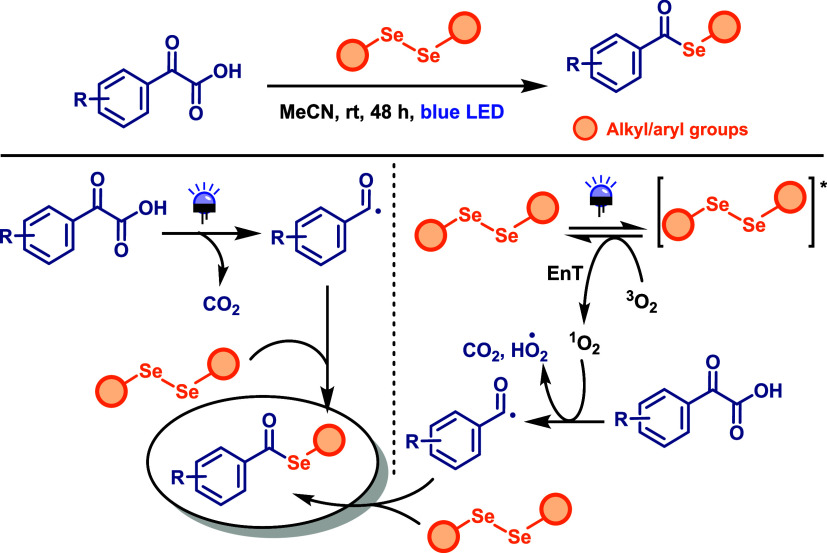
Photocatalyst- and Oxidant-Free Radical Decarboxylative Coupling of α-oxo Acids with Diselenides

## Carbon-Boron Bonds

The chemistry of organoboron compounds has expanded significantly over the past decades, driven largely by the development of the Suzuki–Miyaura reaction. However, traditional methods for their preparation often suffer from limitations related to the instability of the compounds and the poor functional group tolerance. In this context, photocatalytic strategies have emerged as attractive alternatives for borylation reactions. The first example of photochemical direct decarboxylative borylation of aromatic carboxylic acids was reported by Yoshimi and co-workers.^67^ In this protocol, arylboronate esters are formed via photoinduced decarboxylation of benzoic acid derivatives using bis­(pinacolato)­diboron (B_2_pin_2_) as the boron source (Scheme 39). The reaction was carried out in the presence of stoichiometric amounts of 1,4-dicyanonaphthalene (DCN) and biphenyl (BP) under 405 nm LED irradiation for 6 h at 30 °C, using a MeCN/H_2_O (9:1) solvent system. The reaction mechanism begins with the photoexcitation of DCN to its excited state (DCN*), which oxidizes biphenyl to generate the biphenyl radical cation (BP^•+^) along with the reduced DCN radical anion (DCN^•–^). The BP^•+^ species subsequently oxidizes the carboxylate anion, resulting in decarboxylation and formation of an aryl radical. This radical then couples with B_2_pin_2_ to furnish the desired arylboronate product (Scheme 39). The proposed method is operationally simple and offers several environmental advantages, including mild conditions and the absence of metal catalysts. Nonetheless, the reaction exhibits a relatively narrow substrate scope and provides the target products with low to moderate yields.

**Scheme 39:**
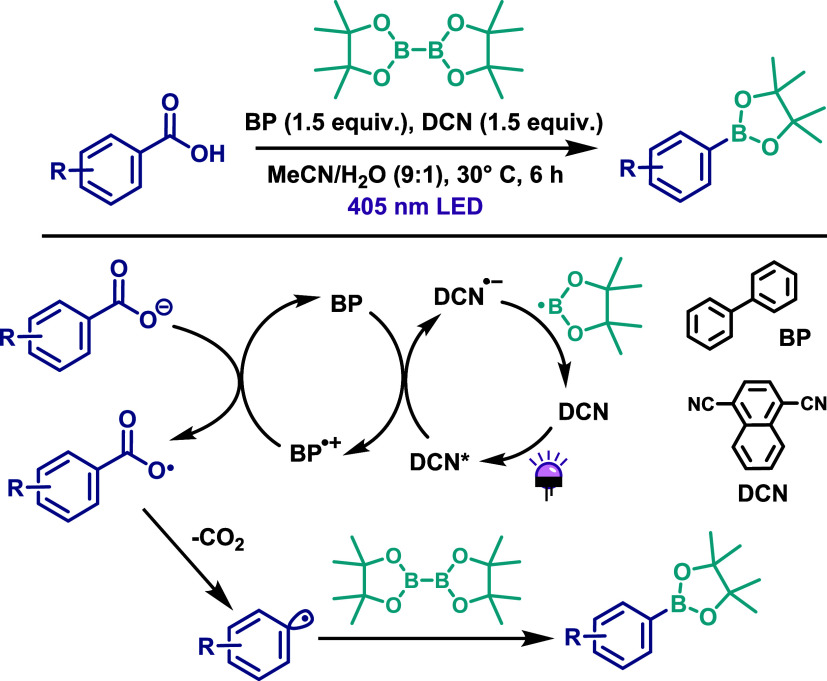
Photoinduced Decarboxylative Borylation of Benzoic Acid Derivatives

A similar procedure was later reported by Liu’s group,^73^ in which the direct photochemical decarboxylative borylation of carboxylic acids was achieved using a photocatalytic approach. The reaction between aryl carboxylic acids and B_2_pin_2_ was performed using a biomimetic design, employing tetramethylguanidine (TMG) as the base and a dual catalytic system consisting of [Ir­(dF­(CF_3_ppy)_2_)­(5,5′-CF_3_-bpy)]­PF_6_ (3 mol %) and Co­(dmgH)_2_pyCl (15 mol %) under 440 nm LED irradiation for 24 h, using ethyl acetate as the solvent (Scheme 40). This protocol enabled the synthesis of a broader range of arylboranate products in moderate to good yields. However, benzoic acids bearing electron-withdrawing groups afforded the corresponding products in only low to moderate yields. Moreover, substrates containing strongly coordinating or oxidizable functional groups (such as amines or phenols) were unreactive. The proposed mechanism begins with the formation of a hydrogen-bonded complex between TMG and the benzoic acid. Previous studies have shown that such complexes can alter the substrate′s redox properties and thereby facilitate single-electron transfer (SET).^74^ In parallel, photoexcitation of the Ir­(III) photocatalyst generates an excited Ir­(III)* species, which reacts with the Co­(III) cocatalyst to produce an Ir­(IV) species capable of oxidizing the TMG complex. The resulting aryl radical, formed via decarboxylation, subsequently couples with B_2_pin_2_ to yield the desired product. Computational studies conducted by the authors revealed that TMG can also form a complex with B_2_pin_2_, which stabilizes the fragmentation product of B_2_pin_2_ and increases the thermodynamic driving force for the radical coupling. Additionally, the presence of an air atmosphere was found to be essential for reoxidizing the Co­(II) cocatalyst back to Co­(III), thereby closing the catalytic cycle (Scheme 40).

**Scheme 40:**
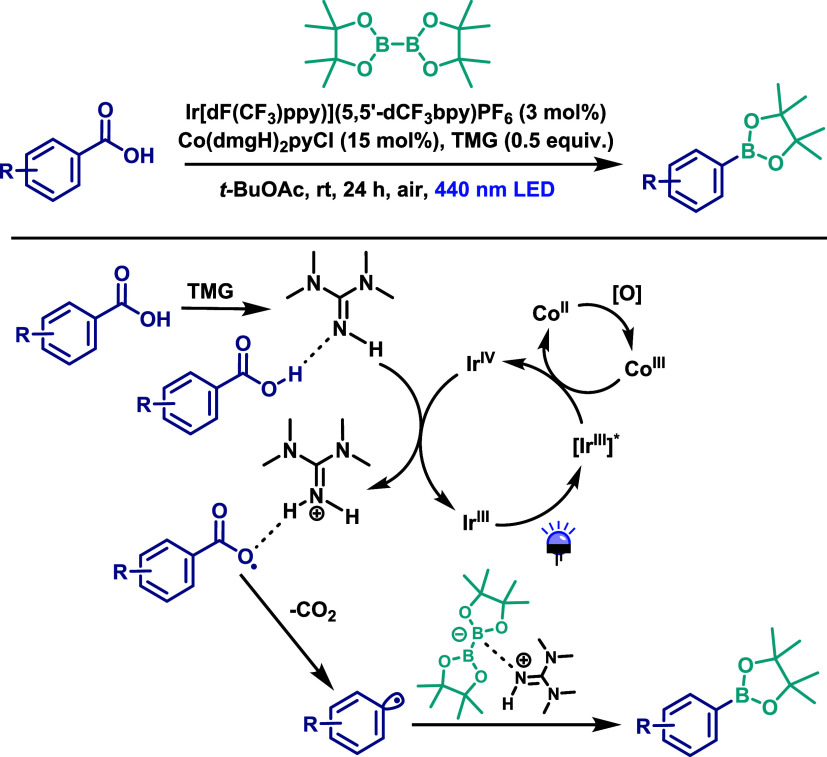
Ir-Catalyzed Decarboxylative Borylation of Carboxylic Acids

Further improvements in the photocatalytic decarboxylative borylation of carboxylic acids were reported by MacMillan’s group through the application of copper charge-transfer catalysis.^75^ In this methodology, aryl carboxylic acids react with B_2_pin_2_ is performed in the presence of [Cu­(MeCN)_4_]­BF_4_ (20 mol %) as the catalyst, NFSI (3 equiv) as the oxidant, and NaF and LiClO_4_ as additives, under 365 nm LED irradiation for 6 h, in MeCN (Scheme 41). The proposed mechanism begins with the formation of a Cu­(I) carboxylate complex, which is oxidized to a Cu­(II) species by NFSI. Upon light absorption, a LMCT process occurs, generating an aryl radical that subsequently couples with B_2_pin_2_ to afford the corresponding arylboronic ester. NaF and LiClO_4_ acts as MeCN-soluble ionic additivies that activate B_2_pin_2_ through the formation of a lithium fluoroborate intermediate (Scheme 41). This protocol proved efficient and broadly tolerant of various functional groups. Notably, the authors also demonstrated a one-pot Pd-catalyzed Suzuki–Miyaura coupling of the in situ-generated boronic ester with aryl bromides, eliminating the need for intermediate purification. The final coupling products were obtained in moderate yields, highlighting the versatility and synthetic utility of the method.

**Scheme 41:**
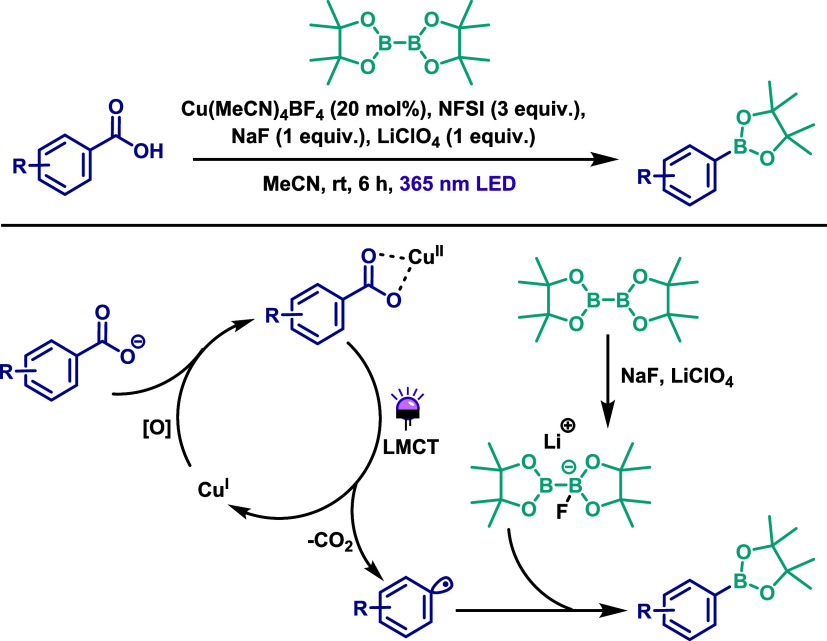
Cu-Catalyzed Decarboxylative Borylation of Carboxylic Acids

## Conclusion

Considering the constant demand for fine synthetic chemicals, photoinduced decarboxylative radical coupling reactions employing free carboxylic acids have recently emerged as an effective and rapidly growing strategy for constructing a variety of chemical bonds beyond the traditional carbon–carbon bond. The wide availability and structural diversity of carboxylic acids make them among the most promising substrates for the future development of synthetically valuable methodologies. Despite the diversity of applications and mechanistic pathways, the photoinduced decarboxylation of free carboxylic acids still requires further exploration to expand the scope of carbon-heteroatom bond formation such as enabling C–Si and C–P bond construction, as well as to improve the yields, applicability, and sustainability of the current protocols. In this context, the use of 3d metals (particularly Fe­(III)) as photocatalysts, or the development of catalyst-free approaches is highly desirable and should be prioritized. Furthermore, scalability studies and the adaptation of current methods to industrially relevant platforms, such as continuous flow systems, remain underexplored in the literature, despite their crucial importance for large-scale applications.
